# Bacteriophages fEV-1 and fD1 Infect *Yersinia pestis*

**DOI:** 10.3390/v13071384

**Published:** 2021-07-16

**Authors:** Mikael Skurnik, Salla Jaakkola, Laura Mattinen, Lotta von Ossowski, Ayesha Nawaz, Maria I. Pajunen, Lotta J. Happonen

**Affiliations:** 1 Department of Bacteriology and Immunology, Medicum, Human Microbiome Research Program, Faculty of Medicine, University of Helsinki, 00290 Helsinki, Finland; salla.jaakkola@hus.fi (S.J.); mattinen.laura@gmail.com (L.M.); mail2ayesha@gmail.com (A.N.); maria.pajunen@helsinki.fi (M.I.P.); 2 Division of Clinical Microbiology, HUSLAB, University of Helsinki and Helsinki University Hospital, 00290 Helsinki, Finland; 3 Department of Medical Biochemistry, University of Turku, 20520 Turku, Finland; l.v.ossowski@gmail.com; 4 Division of Infection Medicine, Department of Clinical Sciences Lund, Lund University, 22184 Lund, Sweden; lotta.happonen@med.lu.se

**Keywords:** bacteriophage, *Yersinia pestis*, dwarf myovirus, myovirus, genome, proteome

## Abstract

Bacteriophages vB_YpeM_fEV-1 (fEV-1) and vB_YpeM_fD1 (fD1) were isolated from incoming sewage water samples in Turku, Finland, using *Yersinia pestis* strains EV76 and KIM D27 as enrichment hosts, respectively. Genomic analysis and transmission electron microscopy established that fEV-1 is a novel type of dwarf myovirus, while fD1 is a T4-like myovirus. The genome sizes are 38 and 167 kb, respectively. To date, the morphology and genome sequences of some dwarf myoviruses have been described; however, a proteome characterization such as the one presented here, has currently been lacking for this group of viruses. Notably, fEV-1 is the first dwarf myovirus described for *Y. pestis*. The host range of fEV-1 was restricted strictly to *Y. pestis* strains, while that of fD1 also included other members of Enterobacterales such as *Escherichia coli* and *Yersinia pseudotuberculosis*. In this study, we present the life cycles, genomes, and proteomes of two *Yersinia* myoviruses, fEV-1 and fD1.

## 1. Introduction

*Yersinia pestis*, the causative agent of bubonic and pneumonic plague, is a zoonotic bacterium spread by fleas in small rodents [1]. Around 2000 cases of human plague are reported to WHO every year, Madagascar leading the statistics with hundreds of cases per year with many leading to death [2,3]. A closely related bacterium, *Yersinia pseudotuberculosis* and the more distant *Yersinia enterocolitica*, cause gastroenteritis and mesenteric lymphadenitis in humans. Antibiotic resistant strains of *Y. pestis* and *Y. pseudotuberculosis* are an emerging threat, and concerns for biological warfare have also been voiced [4]. Due to these threats, managing bacterial infections using bacteriophages, i.e., phage therapy, has gained serious interest lately, also in the case of *Y. pestis* [5]. Several phages that infect *Y. pestis* have been described throughout the years and, recently, these have been reviewed [6]. Indeed, Felix d’Herelle used phage therapy against bubonic plague in the 1920s [7].

*Y. pestis* specific phages, such as ΦA1122 [8] and L-413C [9], have been used in *Y. pestis* diagnostics and identification, and the potential of phage therapy was also studied [5]. For therapeutic use, phages need to be both lytic and nontoxic to humans. Reported lytic *Yersinia* phages are mostly podoviruses [6], but myoviruses, such as PST [10], ΦJA1 [11], PY100 [12], and JC221 [13] have also been described. In addition to infectious “live” phage particles, certain phage proteins can also act as antimicrobials [14].

Members of the Myoviruses are typically dsDNA viruses with isometric heads and contractile tails. *Escherichia virus T4* is the type species in genus *Tequatrovirus* of the subfamily *Tevenvirinae* that belongs in the classification of the International Committee for the Taxonomy of Viruses (ICTV) to the branch *Duplodnaviria > Heunggongvirae > Uroviricota > Caudoviricetes > Caudovirales > Myoviridae* (https://talk.ictvonline.org/taxonomy/ 22 April 2021). Tequatroviruses are lytic and infect Gram-negative bacteria. They typically have elongated heads around 120 × 86 nm in size, an average tail length of approximately 140 nm, and their genomes are fairly large, around 160–250 kb in size [15]. Currently, ICTV classifies two sequenced *Y. pestis* infecting phages, PST and fD1, as their own species in genus *Tequatrovirus* [16].

Myoviruses with small-sized heads and genome sizes below 50 kb have been called “dwarf” myoviruses [17,18]. Their head diameters vary from 55 to 75 nm, and most have tails from 55 to 85 nm, although *Aggregatibacter* phages with 112–115 nm tails have also been included in this group [17]. Some of these viruses share sequence homology and genome synteny, and two different genera have been suggested. The ΦPLPE-like viruses [17] include both lytic and temperate phages, and they infect a wide range of Gram-negative bacteria, whereas the proposed phiMMP04likevirus consists of phages infecting *Clostridium difficile* [19]. There are only two dwarf myoviruses infecting *Yersinia*, i.e., PY100 and fEV-1, that have been isolated so far [6,12].

In this study we present detailed characterization of two phages that infect *Y. pestis.* The phages fEV-1 and fD1 were isolated in 1999 from a Turku City sewage water sample. We originally named the phages ΦEV-1 and ΦD1 or phiD1, but to avoid using the Greek letters, hereafter, we use the names fEV-1 and fD1. Their annotated genome sequences were submitted to sequence databases under accession numbers LT992259 and HE956711, respectively.

## 2. Materials and Methods

### 2.1. Bacterial Strains, Phages, and Media

Bacterial strains and phages used in this study are listed in Table 1. *Yersinia* cultures were grown at room temperature (RT, 22 °C) in tryptone soy broth (TSB, Lab M) and *E. coli* strains at 37 °C in lysogeny broth (LB). For plating, brain heart infusion agar (BHI, Becton Dickinson Co., Oxford, UK) was used for *Yersinia* strains and LB agar (LA) for *E. coli*. For solid plates and soft agar media, 1.5% or 0.4% bacteriological agar (Lab M) was used, respectively. Kanamycin (Kan, 30 µg/mL) was added when required.

### 2.2. Phage Titration, Host Screening, and Efficiency of Plating

Phage titers were determined by double-layer agar method [27]. For host screening, bacteria (Table 1) were plated using the double-layer method, and drops of fD1 and fEV-1 dilutions were added on plates. Plates were incubated overnight and screened for plaques to determine the host specificity. Efficiency of plating was further determined on susceptible hosts by titration. *Y. pestis* D27 and EV76 were used as positive controls.

### 2.3. Phage Production and Purification

For phage production, overnight liquid cultures of *Y. pestis* D27 or *Y. pestis* EV76 were plated with fD1 or fEV-1, respectively, to produce semi confluent plates. The soft agar layer was mixed with SM buffer (100 mM NaCl, 10 mM MgSO_4_, 50 mM Tris-HCl (pH 7.5), 0.01% gelatin), centrifuged, and the supernatant was treated with chloroform to lyse any remaining bacteria. The lysate was filtered, and sucrose was added to a final concentration of 0.8% (*w*/*v*).

The phage preparations were further purified by discontinuous glycerol density gradient ultracentrifugation through 5% and 40% glycerol layers in TM buffer using the Beckman BSW55Ti rotor (Indianapolis, IN, USA) at 40,000 rpm at 4 °C for 3 h [28]. The phage pellet was resuspended in SM buffer containing 8% sucrose.

### 2.4. Electron Microscopy

Aliquots of phage preparate (5 μL) were adsorbed on holey-carbon film coated grids (Quantifoil R 2/2, Electron Microscopy Sciences, Hatfield, PA, USA) for 1 min prior to negative staining with 2% (*w*/*v*) uranyl acetate (pH 4.5). The specimens were imaged in a FEI Tecnai F20 field emission gun transmission electron microscope (FEI, Eindhoven, the Netherlands) operating at 200 kV. Micrographs were recorded on a Gatan UltraScan 4000 charge-coupled-device (CCD) camera (Gatan, Inc., Pleasanton, CA, USA) at nominal magnifications of 39,440× and 68,000×. The data were collected in the Biocenter Finland National Cryo-EM unit, Institute of Biotechnology, University of Helsinki (Helsinki, Finland).

### 2.5. Phage Genome Extraction, Sequencing, and Analysis

Phage DNA was obtained from high-titer phage preparations, as described earlier [27]. Phage fD1 DNA was sequenced using Illumina GAIIx (genome analyzer) technology at the FIMM Sequencing unit (Helsinki, Finland). The sequence assembly was performed with the NextGene (http://www.softgenetics.com (accessed on 12 December 2010)) and Staden software packages [29]. The Artemis genome-browsing and annotation tool [30] was used for genome annotation. Phage fEV-1 DNA was sequenced as above but due to low quality, complemented in addition with a second round using the sequencing service at Novogene Europe (Cambridge, UK) using Illumina HiSeq (San Diego, CA, USA) with 150 bp paired end reads.

The obtained sequence reads were assembled de novo using the A5-miseq pipeline [31]. The read coverages of the resulting contigs were checked with the Artemis software [32,33] and contaminating bacterial genomic sequences identified by their >100-fold lower read coverages (the read coverages of the phage contigs were >10,000) and BLASTN searches against the *Y. pestis* CO-92 genome sequence (accession no. CP009973). To verify the fidelity of the assemblies, the reads were mapped back to the de novo assembled contigs using the tools of the Geneious Prime software version 2021.0.3 (Biomatters Ltd., Auckland, New Zealand) [34]. Preliminary annotations of the phage genomes were carried out using Rapid Annotation Subsystems Technology [RAST] [35] that was manually checked and revised with the Artemis software [32,33]. The PhageTerm program was used to identify the termini of the phage genomes [36] and tRNAscan-SE v. 2.0 was used to identify tRNA genes [37,38].

The identities and functions of the predicted genes and gene products were analyzed using the PSI-BLAST (https://blast.ncbi.nlm.nih.gov/Blast.cgi (accessed on several occasions since 10 February 2012)) server. Different EMBOSS sequence analysis tools were used through the Chipster platform [39] at the Centers for Scientific Computing (https://www.csc.fi/ (accessed on several occasions since 10 February 2012)). The phylogenetic phage proteomic trees were generated by VIPTree [40]. The promoters and terminators were predicted using the BPROM and FindTerm tools [41].

### 2.6. PCR and Sanger Sequencing

The PCR primers (Table 2) were designed using the EMBOSS Eprimer3 tool in Chipster [39], and commercially synthetized at Metabion International AG (Steinkirchen, Germany). The PCRs were performed in 0.2 mL thin-walled PCR tubes (4titude^®^ Ltd., Wotton, Surrey, UK), in a total volume of 25 μL containing 1 μL of DNA template, 0.2 μM of each primer (Table 2), 200 μM of dNTP mix (Thermo Fisher Scientific, Waltham, MA, USA), 2.5 μL of 10× Standard Taq Reaction Buffer, and 1.25 U of Taq DNA Polymerase (Thermo Fisher Scientific, Waltham, MA, USA). The PCR cycling included an initial denaturation at 95 °C for 3 min, followed by 34 cycles each consisting of denaturation at 95 °C for 30 s, 30 s at annealing temperature, and extension for 30 s at 72 °C. This was followed by a final extension step at 72 °C for 5 min, after which PCR products were kept on hold at 4 °C until further processing. The annealing temperatures were calculated using the on-line service at https://www.thermofisher.com/ (accessed on 8 November 2020). The PCR products were analyzed using 1% agarose gel electrophoresis and cleaned using the Nucleospin Gel extraction and PCR clean-up kit (Macherey-Nagel GmbH, Düren, Germany). The purified PCR fragments were sequenced with appropriate sequencing primers using the Sanger sequencing service at the Institute for Molecular Medicine Finland (https://www.fimm.fi/en/services/technology-centre/sequencing (accessed on 4 December 2020)).

### 2.7. Restriction Endonuclease Analysis

Restriction digestions were carried out using the restriction enzymes EcoRI, HincII, NsiI, PstI, SalI, ScaI, SexAI, and SpeI (Thermo Fischer Scientific). These enzymes gave several well separated bands when in silico digested by the NEBcutter (http://nc2.neb.com/NEBcutter2/ (accessed on 8 May 2021)). The digestions were carried out in a final volume of 10 µL, containing DNA (ca. 300 ng), 0.5 µL of enzyme, and 1 µL of Fast digest green buffer (10×, Thermo Fisher Scientific). After 2–16 h incubation at 37 °C, the restriction fragments along with undigested DNA and GeneRuler 1 kb plus DNA Ladder (Thermo Fisher Scientific) were separated on 1% (*w*/*v*) agarose gel and detected with ethidium bromide staining using UV transillumination. Images were recorded using the BioRad GelDoc XR+ imaging system.

### 2.8. Sample Preparation for Mass Spectrometry

For the proteome analyses, the phages fEV-1 and fD1 were purified as described above, except that the final resuspension after ultracentrifugation was done in SM buffer without sucrose. The purified fEV-1 phage preparation was digested for mass spectrometry in triplicates, whereas the fD1 dataset consisted of two different phage preparations both as triplicate samples. Briefly, the samples were mixed with 8 M urea and 100 mM ammonium bicarbonate, and the cysteine bonds were reduced with 5 mM TCEP (37 °C for 30 min) and alkylated with 10 mM iodoacetamide (22 °C for 60 min). Samples were diluted with 100 mM ammonium bicarbonate to a final urea concentration of 1.5 M, and sequencing grade trypsin (Promega, Madison, WI, USA) was added for protein digestion (37 °C for 18 h). Samples were acidified (to a final pH 3.0) with 10% formic acid, and the peptides subsequently purified with C18 reverse phase spin columns, according to the manufacturer’s instructions (Microspin and Macrospin columns, Harvard Apparatus, Holliston, MA, USA). Peptides were dried using a Speedvac and reconstituted in 2% acetonitrile/0.2% formic acid prior to mass spectrometric analyses.

### 2.9. Sample Preparation for Mass Spectrometry

The peptide analyses were performed on a Q Exactive Plus (fEV-1 and fD1) or Q Exactive HFX (fD1) mass spectrometer (Thermo Scientific). The Q Exactive Plus mass spectrometer was connected to an EASY-nLC 1000 ultra-high-performance liquid chromatography system (Thermo Scientific). The peptides were separated on an EASY-Spray column (Thermo Scientific) ID 75 μm × 25 cm, column temperature at 45 °C. The column was equilibrated, and the samples loaded using a constant pressure of 600 bars. The peptides were separated using a linear gradient from 5 to 35% acetonitrile in aqueous 0.1% formic acid for 75 min at a flow rate of 300 nL min^−1^. One full MS scan (resolution 70,000 at 200 *m*/*z*, mass range 400–1600 *m*/*z*) was followed by MS/MS scans (resolution 17,500 at 200 *m*/*z*) of the 15 most abundant ion signals. Precursor ions were isolated with 2 *m*/*z* isolation width and fragmented using higher-energy collisional-induced dissociation (HCD) at a normalized collision energy (NCE) of 30. Charge state screening was enabled, and precursors with an unknown charge state and singly charged ions were rejected. The automatic gain control was set to 1 × 10^6^ for both MS and MS/MS with ion accumulation times of 100 and 60 ms, respectively. The intensity threshold for precursor ion selection was set to 1.7 × 10^4^.

The Q Exactive HFX mass spectrometer was connected to an EASY-nLC 1200 ultra-high-performance liquid chromatography system (Thermo Scientific). Peptides were separated on an EASY-Spray column (Thermo Scientific) ID 75 μm × 25 cm, column temperature 45 °C, operated at a constant pressure of 800 bar. A linear gradient from 5% to 35% acetonitrile in aqueous 0.1% formic acid was run for 65 min at a flow rate of 300 nL min^−1^. One full MS scan (resolution 60,000@200 *m/z*, mass range 350–1400 *m*/*z*) was followed by MS/MS scans (resolution 15,000@200 *m/z*) of the 15 most abundant ion signals. The precursor ions were isolated with 1.3 *m*/*z* isolation width and fragmented using HCD at an NCE of 28. Precursors with an unknown charge state, singly charged ions or with a charge state of 6 or above, were rejected. The dynamic exclusion window was set to 10 s. The automatic gain control was set to 1 × 10^6^ for both MS and MS/MS, with ion accumulation times of 100 and 60 ms, respectively. The intensity threshold for precursor ion selection was set to 1.7 × 10^4^.

### 2.10. Mass Spectrometry Data Analysis

MS raw data were converted to gzipped and Numpressed [42] mzML using the tool msconvert from ProteoWizard, v3.0.5930 suite [43]. All data analyses were stored and managed using openBIS [44]. For fEV-1, acquired spectra were analyzed using the search engine X! Tandem (2013.06.15.1-LabKey, Insilicos, ISB) [45] against an in-house compiled dataset containing the reference proteome of *Y. pestis* CO-92/Biovar Orientalis (UniProt proteome ID UP000000815) and that of *Yersinia* phage fEV-1 (UniProt proteome ID UP000274108) (both accessed on 8 June 2021), yielding a total of 3966 protein entries and an equal amount of reverse decoy sequences. We also performed an additional analysis in order to identify any expressed open reading frames (ORFs) missed in the genome annotation. For this, the genome of fEV-1 was analyzed for ORFs via the NCBI ORF finder tool (https://www.ncbi.nlm.nih.gov/orffinder/ (accessed on 12 June 2021)) using 75 nt as the minimal ORF length, standard genetic code as code, and ATG and alternative initiation codons as ORF start codon. This approach generated 534 translated ORFs, which were used together with an equal amount of reverse decoy sequences as an alternative reference proteome.

For fD1, acquired spectra were analyzed against an in-house compiled dataset containing the *Y. pestis* CO-92/Biovar Orientalis and *Yersinia* phage fD1 reference proteomes (UniProt proteome IDs UP000000815 and UP000002906, respectively), yielding a total of 4186 protein entries and an equal amount of reverse decoy sequences. We also performed the same analysis as for fEV-1 in order to identify any expressed ORFs missed in the original genome annotation. For this, the genome of fD1 (accession number HE956711.1) was analyzed for ORFs via the NCBI ORF finder tool (https://www.ncbi.nlm.nih.gov/orffinder/ (accessed on 12 June 2021)) using 75 nt as the minimal ORF length, standard genetic code as code, and ATG and alternative initiation codons as ORF start codon. This approach generated 505 translated ORFs, which was used together with an equal amount of reverse decoy sequences as an alternative reference proteome.

For both phages, fully tryptic digestion was used allowing one missed cleavage; Carbamidomethylation (C) was set to static and oxidation (M) to variable modifications. Mass tolerance for precursor ions was set to 20 ppm, and for fragment ions to 50 ppm. Identified peptides were processed and analyzed through the Trans-Proteomic Pipeline (TPP v4.7 POLAR VORTEX rev 0, Build 201403121010) using PeptideProphet [46]. A protein was only considered identified, if we could detect peptides in all three replicates with an average spectral count of 2 or more. The mass spectrometry data have been deposited to the ProteomeXchange [47] consortium via the MassIVE partner repository (https://massive.ucsd.edu/ (accessed on 19 June 2021)) with the dataset identifiers PXD026811 (fEV-1) and PXD026812 (fD1).

### 2.11. Phage Growth Curves

Liquid cultures of host bacteria (*Y. pestis* D27 or *E. coli* 538 for fD1, and *Y. pestis* EV76 for fEV-1) in logarithmic phase were infected with a MOI of 0.01 and incubated for 5 min at RT. The remaining free virions were removed by centrifugation (1923× *g* for 3 min) and rinsing the pellet with TSB, and infected cells were resuspended to original volume. Phage titer was determined at different time points to determine the phage latency period and burst size. The measurements were repeated at least three times.

## 3. Results and Discussion

### 3.1. Electron Microscopy

**Figure 1:**
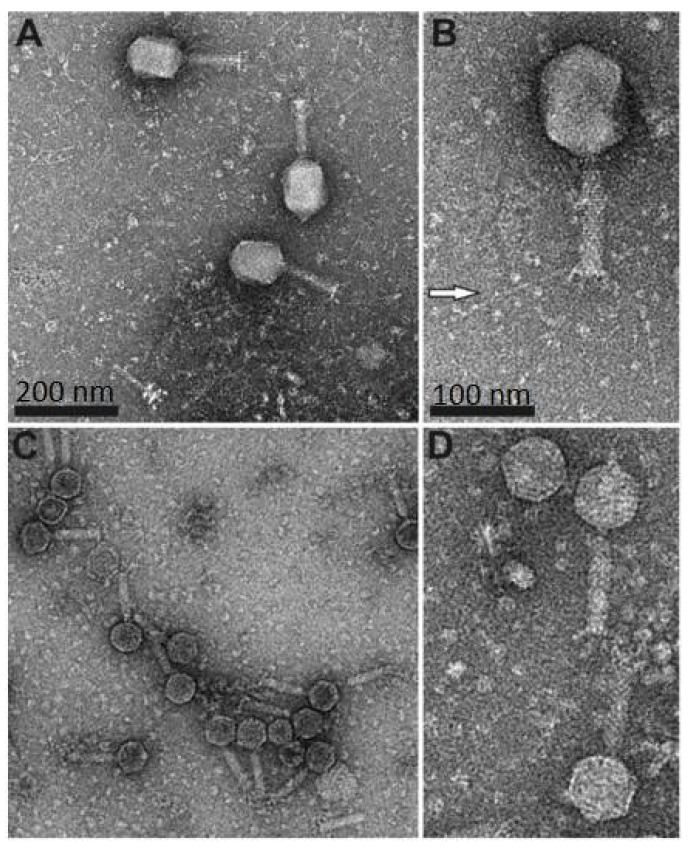
Transmission electron microscopy analysis of phage fD1 (**A**,**B**) and fEV-1 (**C**,**D**) particles. The scale in (**A**) and (**C**) is 200 nm, and in (**B**) and (**D**), 100 nm. White arrow in (**B**), points to one of the long tail fibers of fD1.

Electron microscopy revealed that both fD1 and fEV-1 were tailed phages with a myovirus morphotype. The isometric head of fEV-1 is 65 ± 3 nm vertex-to-vertex, 59 ± 4 nm edge-to-edge, and the tail has an average length of 92 ± 5 nm (*n* = 8 for all measurements). The head measures of fEV-1, hence, align well with the head sizes (55–75 nm) of other dwarf myoviruses, whereas the tail of fEV-1 is slightly longer than the reported 55–85 nm [17,18,48,49]. We do not see any apparent long tail fibers in our fEV-1 preparation; however, several thick and short fibers are clearly visible (Figure 1). The prolate head of fD1 is 111 ± 5 nm long and 83 ± 3 nm wide. The tail measures 112 ± 2 nm with extended long tail fibers of an average length of 135 ± 13 nm (for all fD1 measurements, *n* = 5). These values are very close to those of T4, with a 120 nm long and 86 nm wide prolate head [50], and a 140 nm long contractile tail with six long tail fibers [51].

### 3.2. Phage Host Specificity and Efficiency of Plating

**Figure 2:**
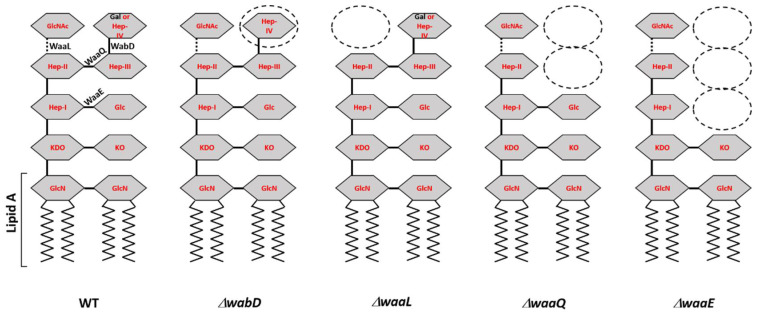
Schematic presentation of the *Y. pestis* LPS mutants used in this study (Table 1). Indicated for the wild type (WT) LPS, are the glycosidic bonds formed by the eliminated glycosyltransferases of the LPS mutants and the part that forms the lipid A. The zigzag lines represent the acyl chains of the lipid A. In the mutant LPS structures, the affected sugar residues are indicated by dashed ovals. Gln, D-glucosamine; KDO, 3-deoxy-D-*manno*-oct-2-ulopyranosonic acid; KO, D-*glycero*-D-*talo*-oct-2-ulopyranosonic acid; Hep-I, Hep-II, and Hep-III, L-*glycero*-D-*manno*-heptopyranose; Hep-IV, D-*glycero*-D-*manno*-heptopyranose; Glc, D-glucopyranose; Gal, D-galactopyranose.

All *Y. pestis* laboratory strains, and some clinical strains of *E. coli* (storage #s 6588, 6589, and 6590) and *Y. pseudotuberculosis* (#s 2069, 2070, and 2649) were susceptible to fD1 (Table 1). Of the tested strains, the fD1 efficiency of plating (Table 1) was highest on *E. coli* strain 6588, which was later used in the growth curve experiment (see below). While fD1 infected equally all the different *Y. pestis* D27 LPS-mutants (Figure 2) with progressively truncated LPS core structures [21], for fEV-1, there were some differences in plating efficiencies (Table 1). The EOP of fEV-1 on the Δ*waaQ* mutant was 4 logs lower than in LPS wild type strain indicating that the heptose III and IV residues, missing in the mutant, were essential components of the fEV-1 receptor. Furthermore, the additional truncation of the structure by the heptose I -linked glucose in the Δ*waaE* mutant decreased the EOP more, altogether 5 logs, indicating that the glucose residue is also an essential component of the receptor. In the Δ*waaE* mutant, fEV-1 formed small and turbid plaques that were difficult to see. Interestingly, the distal GlcNAc residue eliminated in the Δ*waaL* mutant appeared to play no role for the fEV-1 receptor.

### 3.3. Phage Growth Curves

**Figure 3:**
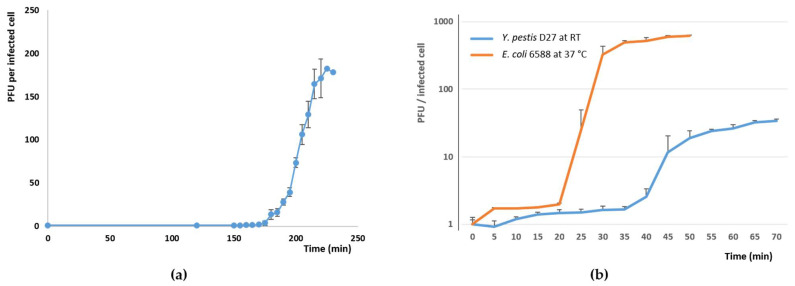
One-step growth curves for the phages: (**a**) Growth curve of fEV-1 on *Y. pestis* EV-76 at RT; (**b**) growth curves of fD1 on *Y pestis* D27 at RT and on *E. coli* 6588 at 37 °C. Note the logarithmic scale in (b) that allowed the use of only the positive SD bars.

**Figure 4:**
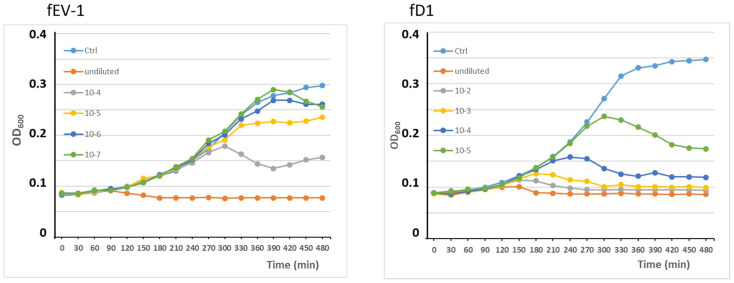
Growth of *Y. pestis* strains EV-76 and D27 infected with different dilutions of phages fEV-1 and fD1, respectively, followed by OD_600_ measurements every 30 min for 8 h.

In the one step growth curve of phage fEV-1, grown on *Y. pestis* EV76 at RT, the latency period was exceptionally long, i.e., 185 min, with a burst size of >180 plaque forming units (PFU) per infected cell (Figure 3a). In comparison, the dwarf myovirus ΦPLPE has a 90 min latent period and a burst size of 100 particles [52]. The fEV-1 latency period was twice as long, and the burst size bigger. It is possible that dwarf myoviruses, in general, have long latency periods, but very scarce data has been published on that topic. Moreover, for ΦPLPE, a long latency due to the host being lysogenized by the phage or already harboring a temperate ϕPLPE giving the host superinfection immunity, has been ruled out [52]. In *Y. pestis* D27 grown at RT, fD1 had a latent period of 35–40 min and had a burst size of 20–30 PFU per infected cell at 50–60 min. On the contrary, when grown in *E. coli* # 6588 at 37 °C, the growth was significantly more vigorous with a short 20 min latency period and a burst size of >500 PFU per infected cell at 35 min after infection (Figure 3b). The phages both appeared to be efficient in killing *Y. pestis* (Figure 4). In the fEV-1 infected cultures, regrowth of bacteria, indicating appearance of phage-insensitive mutants, started after 6–7 h of incubation. Similar phenomenon was not apparent for fD1-infected cultures.

### 3.4. The Genome and Taxonomic Position of fEV-1

**Figure 5:**
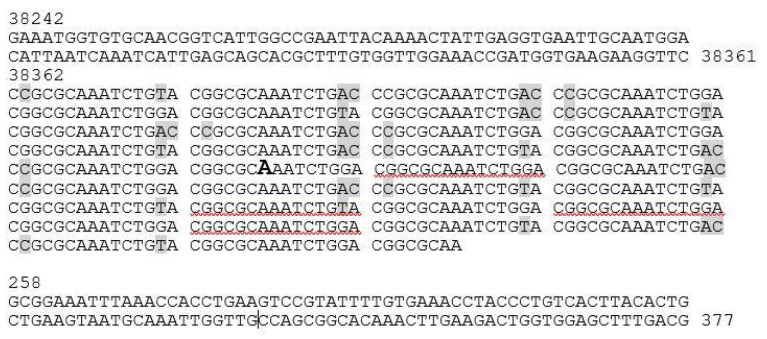
fEV-1, repeat region spanning the nucleotide position 1. The numbers refer to the nucleotide positions in the genome sequence (accession no. LT9922259). The oversized bold A indicated the arbitrarily chosen nucleotide 1 of the sequence.

The genome of fEV-1 is small, only 38,622 bp in size; smaller than that of several other dwarf myoviruses [17]. Altogether, four, eleven, seven, and seven restriction sites for AvaI, EcoRV, NcoI, and SexAI, were identified from the sequence, respectively. Restriction digestions with these enzymes produced the in silico predicted restriction fragments, but did not allow unambiguous identification of the physical termini of the genome (Figure S1). PhageTerm analysis could neither identify any fixed termini (Figure S2). Inspection of the sequences at the predicted ends of the genome revealed the presence of a long repeat region that the NGS approach failed to assemble correctly. Several primers were designed on both sides outside the repeat region (Table 2) and the sequences of the fragments amplified by PCR were determined by Sanger sequencing. The results showed that the repeat region was 518 bp long and contained, altogether, 34.5 repeats of the 15 bp sequence 5′-C(g/c)GCGCAAATCTG(g/t/a)(a/c)-3′ that spanned the repeat region. At that point, we had annotated the nucleotide 1 inside the repeat region (Figure 5), but the proteome analysis carried out later revealed that the repeat region was within a coding sequence encoding a pentapeptide-repeat-containing protein (see Section 3.5). Altogether, these findings suggested that the genome might be circularly permuted. This conclusion was supported by an in silico prediction of the restriction fragments, assuming that the genome is circular (Figure S1), which perfectly matched the experimental data.

**Figure 6:**
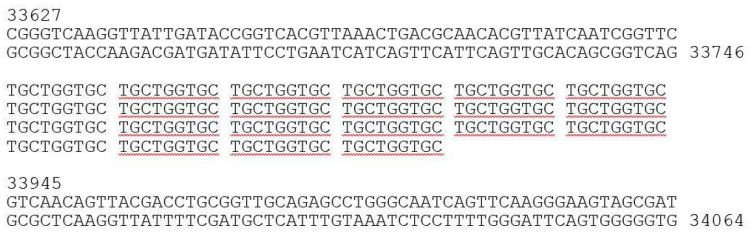
fEV-1, the repeat region within gene *g51c* encoding a hypothetical protein with 22 Pro-Ala-Ala repeats. The numbers refer to the nucleotide positions in the genome sequence (accession no. LT9922259).

Another repeat region of 198 bp was located at 33 kb of the genomic map within the gene *g51c* (Figure 6). It contained 22 repeats of the 9 bp sequence 5′-TGCTGGTGC-3′, thereby, encoding a similar number of Pro-Ala-Ala repeats for Gp51c. In the proteome analysis (see below), Gp51c was detected as a phage particle associated protein (PPAP). However, the role of the Pro-Ala-Ala repeats for Gp51c remains unknown.

**Figure 7:**
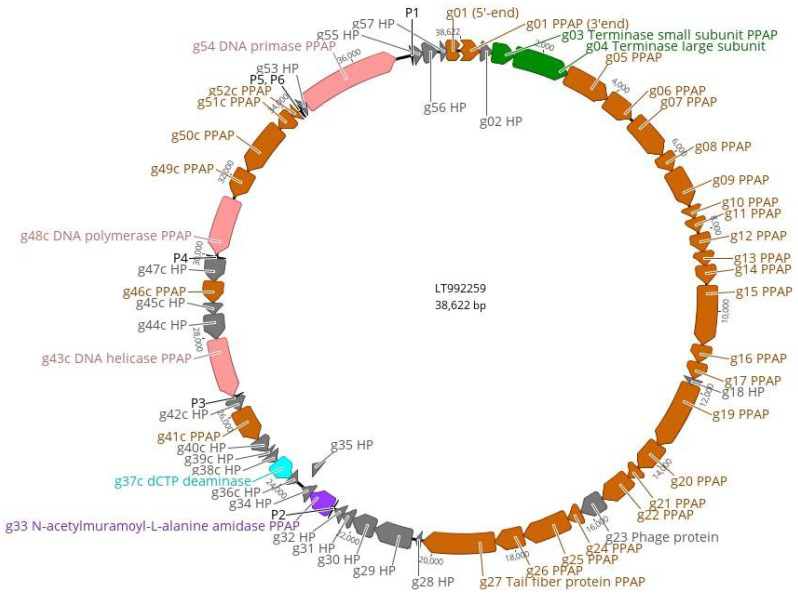
Genomic map of phage fEV-1. The predicted genes are indicated by arrows, and different colors are used to indicate functional classes of the predicted gene products. Brown, structural and phage particle associated proteins (PPAP); turquoise, nucleotide metabolism; green, DNA packaging; violet, lysis; pink, DNA replication and repair; grey, hypothetical proteins (HP). The predicted sigma70-like promoters P1–P6 (Table S1) are indicated by black arrowheads. The figure was generated using Geneious 10.2.6 (www.geneious.com (accessed on 14 July 2021)).

These results allowed us to finalize and annotate the genomic nucleotide sequence of fEV-1. The data were complemented by the proteomics data to identify the PPAPs (Section 3.5), and by the prediction of six sigma70-like promoters (P1–P6, Table S1). The overall organization of the genome is presented in Figure 7.

Altogether, 57 predicted genes were identified from the genome with 40 genes in the forward and 17 genes in the reverse strand organized in three different blocks. The promoters P1, P2, and P6 initiate transcription in the forward direction, and the promoters P3, P4, and P5 initiate the transcription of the reverse strand (Figure 7). According to the LC-MS/MS analysis (see below), 33 gene products were identified as PPAPs and 24 as hypothetical proteins (HPs). The nonstructural gene products with a predicted function included the DNA polymerase (Gp48c), DNA primase (Gp54) and DNA helicase (Gp43c), the small and large terminase subunits (Gp03 and Gp04), *N*-acetylmuramoyl-L-alanine amidase (Gp33), and deoxycytidine triphosphate deaminase (Gp37).

**Figure 8:**
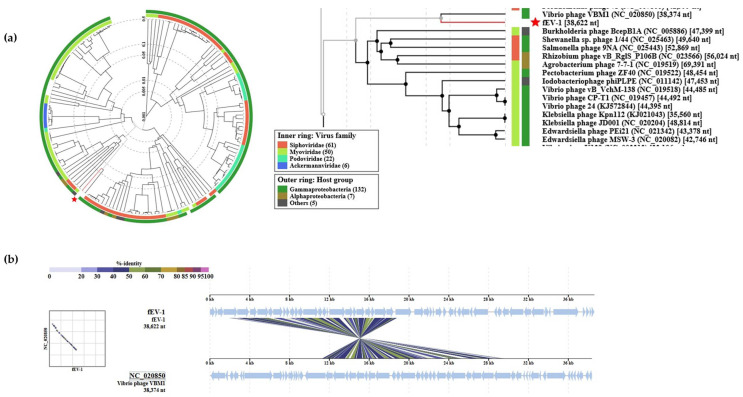
Position of fEV-1 in the Phage Proteomic Tree generated by VIPTree [40]: (**a**) At left, a circular proteomic tree of prokaryotic dsDNA viruses colored by indicated virus families and host taxonomic groups, at right, part of the rectangular presentation of the proteomic tree showing the closest related phages to fEV-1. The location of fEV-1 in both is indicated by a red asterisk; (**b**) the genomic alignment of phages fEV-1 and VBM1.

The VIPtree analysis was carried out for fEV-1 and the results are shown in Figure 8. The analysis positioned fEV-1 next to the *Vibrio* phage VBM1 (accession no. NC_020850) and close to the number of Myoviruses with 35–70 kb genomes (Figure 8a). The similarity with VBM1 spans a 16 kb region and is not very high (Figure 8b), indicating that fEV-1 clearly represents a new dwarf myovirus type, which, at present, is classified as an unclassified myovirus in the taxonomical branch *Duplodnaviria > Heunggongvirae > Uroviricota > Caudoviricetes > Caudovirales > Myoviridae*.

### 3.5. The Proteome of fEV-1

We used in-solution tryptic digestion of fEV-1 virions purified by ultracentrifugation to identify expressed viral proteins associated with the virion. By using label-free data dependent acquisition (DDA) quantification, and two different in-house generated datasets for peptide matching, altogether, we identified 32 viral and 240 host-derived proteins associated with the virion at a false discovery rate (FDR) of <1% (ProteomeXchange dataset PXD026811). The identified fEV-1 proteins constitute 56% of the 57 gene products predicted for fEV-1 (Table S2). According to the label-free quantification, the most abundant PPAP was Gp09, which is closely related to the major capsid protein of the *Vibrio* phage 1.052.A._10N.286.46.C3 (Table S2) [53]. Five fEV-1 predicted gene products had no known homologs in the databases based on PSI-BLAST analysis (Table S2).

One of the in-house generated datasets contained the fEV-1 and the *Y. pestis* CO-92/Biovar Orientalis proteomes, and the other dataset contained all six frame translations of the fEV-1 genome, for a total of 534 translated ORFs. This approach allowed us to detect that we had missed the 5′-end of the gene *g01* overlapping the predicted physical ends of the genome and that the predicted gene started at nucleotide 38297. The revised *g01* is 837 bp in size, encoding a 279 amino acids long gene product homologous to many bacterial pentapeptide repeats containing proteins of *Pseudomonas, Salmonella, Raoultella, Cronobacter,* and *Rhizobium* including the type III secretion system effector protein PipB2 of *Salmonella* [54] and the *Caulobacter* podovirus Jess A (GenBank QCW21951.1). This raised the number of PPAPs to 33. Importantly, our results confirm the expression of more than 20 uncharacterized PPAPs (Table S2), warranting further biochemical characterization of their functions and role in virus replication and assembly.

### 3.6. The Genome and Taxonomic Position of fD1

**Figure 9:**
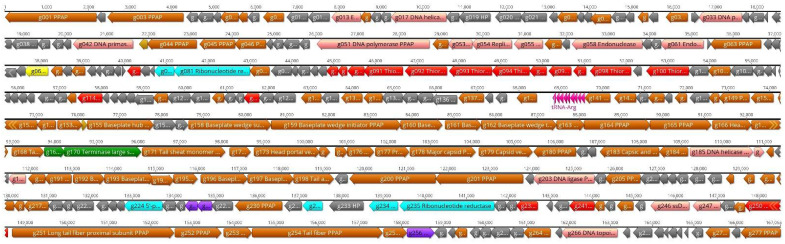
The genomic organization of phage fD1. The predicted genes are indicated by arrows, and different colors are used to indicate functional classes of the predicted gene products. Brown, structural and phage particle associated proteins (PPAP); turquoise, nucleotide metabolism; yellow, transcription; green, DNA packaging; violet, lysis; pink, DNA replication and repair; red, auxiliary metabolism; grey, hypothetical proteins (HP); lilac, tRNA genes. The figure was generated using Geneious 10.2.6 (www.geneious.com (accessed on 14 July 2021)).

The genome of fD1 is 167,063 bp in size and contains 277 predicted protein coding and 9 tRNA genes (Figure 9).

**Figure 10:**
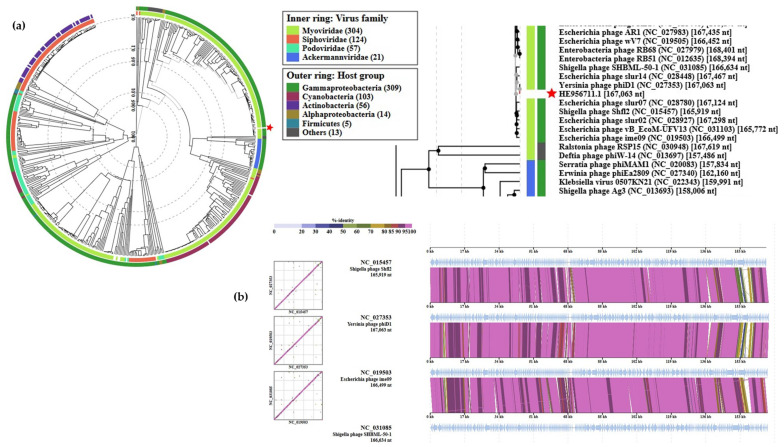
Position of fD1 in the Phage Proteomic Tree generated by VIPTree [40]: (**a**) At left, a circular proteomic tree of prokaryotic dsDNA viruses colored by indicated virus families and host taxonomic groups, at right, part of the rectangular presentation of the proteomic tree showing the closest related phages to fD1. The location of fD1 in both is indicated by a red asterisk; (**b**) Alignment of fD1 genome with its closest related phages.

The phages closest related to fD1 were found among several closely related *Escherichia* and *Shigella* phages (Figure 10). fD1 shows a maximum of 91% overall identity with *Shigella* phage Shfl2 (NC_015457). ICTV classifies fD1 as the only representative of phage species *Yersinia virus D1* that belongs in the taxonomy to the *Duplodnaviria* > *Heunggongvirae* > *Uroviricota* > *Caudoviricetes* > *Caudovirales* > *Myoviridae* > *Tevenvirinae* > *Tequatrovirus* branch.

### 3.7. The Proteome of fD1

We used in-solution tryptic digestion of fD1 virions purified by ultracentrifugation to identify expressed viral proteins associated with the virion from two different virus preparations. By using label-free DDA quantification and two different in-house generated datasets for peptide matching, we identified several viral and host-derived proteins associated with the virion at a false discovery rate (FDR) of <1% (ProteomeXchange dataset PXD026812). Altogether 113 fD1 gene products were identified as PPAPs (Table S3) using a threshold of two or more peptides identified per protein, at an average spectral count of two or more. A total of 70 proteins were identified in both preparations, whereas 20 and 23 proteins were identified in only one of the preparations, respectively. Importantly, one of our in-house generated datasets contained all six frame translations of the fD1 genome, yielding 505 translated ORFs. This approach allowed us to detect a total of nine coding regions originally missed in the original annotation of the fD1 genome. These predicted genes encode for the gene products Gp8, Gp14, Gp29, Gp31, Gp45, Gp51, Gp55, Gp56, and Gp63. If we include identified proteins falling below the stricter threshold above (see Table S3 for details), there are another 17 PPAPs. Homologues to some of these gene products (Gp147, Gp160, Gp167, Gp169, Gp191, Gp199, Gp202, Gp215, and Gp233) are expressed by *Escherichia* phage T4, indicating that they might be true PPAPs, despite falling below our stricter thresholds. Other PPAPs falling below the threshold are located within an operon (Table S3), again, indicating that they most likely are true PPAPs. Notably, for fEV-1, these same less strict settings did not yield additional identifications. If we include all 137 identified fD1 proteins, they constitute 49% of the 277 gene products predicted for fD1. In addition to expressed fD1 gene products, we identified 672 co-purifying host proteins, 372 of which were identified in both virus preparations, and the remaining in only either preparation. Importantly, our results confirm the expression of close to 40 uncharacterized PPAPs (Table S3) warranting further biochemical characterization of their functions and role in virus replication and assembly.

We recently characterized three T4-like *Yersinia* phages, i.e., fPS-2, fPS-65, and fPS-90 [55], and, as phage fD1 is very closely related (87% genome identity) to them, we wanted to compare their long tail fiber (LTF) proteins as they are the ones that first recognize the bacterial host surface [56]. In T4-like phages, the LTFs are typically composed of four subunits, identified as Gp34–Gp37 for T4. Additionally, in T4, Gp38 functions as a chaperone that guides the Gp37 folding and trimerization, and does not remain associated with the LTF after its assembly [57]. In the LTF structure, Gp34 is proximal to the tail baseplate, followed by Gp35 and Gp36 that form the hinge to which is attached the trimeric Gp37. In phage T4, the distal tip of Gp37 is the adhesin, while in T2 type phages, the adhesin is the Gp38 chaperone that after helping the Gp37 trimerization remains associated to the tip of LTF [57].

We identified the fD1 genetic locus that encompasses genes *g251*–*g255*, as those encoding for the long tail fiber subunits. Comparison of the fD1 locus to those of the fPS phages revealed that they shared practically no similarity. As the fPS phages were proposed to utilize, similar to *Salmonella* phage S16 [57], the phage T2 type Gp38 chaperone homolog as the receptor binding protein [56], we carried out BLAST searches to identify closest related homologs for the Gp251–Gp255 of fD1.

The fD1 genes revealed extensive similarity to the corresponding genes of *Escherichia* phages Mobillu (accession no. MN850622.1), vB_EcoM_WFbE185 (MK373778.1), vB_EcoM_KAW3E185 (MK373782.1), vB_EcoM_MM02 (MK373784.1), and *Shigella* phage SHSML-52-1 (KX130865.1), among others. Among the long tail fiber subunits, Gp251, the predicted proximal subunit of the long tail fiber, was 97–99% identical to the homologs of closest *Escherichia*, *Citrobacter*, *Enterobacteria*, *Salmonella* and *Shigella* phages. Gp252, the putative proximal hinge connector protein, and Gp253, the putative distal hinge connector protein of long tail fiber, were both >99% identical to those of several *Escherichia* and *Shigella* phages. Finally, Gp255, the putative tail fiber assembly protein, was 100% identical to homologs of several *Escherichia* phages, indicating that it would not function as a receptor binding protein. On the contrary, Gp254, a 1038 amino-acid-residue LTF distal subunit protein, was significantly less similar to its homologs, showing at best 93% identity to several *Escherichia* phage counterparts, including Mobillu, vB_EcoM_WFbE185, *Shigella* phage SHSML-52-1, among others. Multiple alignment of the sequences revealed that the N-terminal end of the protein was highly conserved between the phages, whereas there were more differences among amino acid residues from 520 to 700. This was followed by a high identity range (residues 701–922), where the identity abruptly broke for about 40 residues followed by 100% identical last 70 C-terminal residues. It is very likely that the last 110 residues make up an intramolecular chaperone (IMC) that is auto cleaved away after the trimerization of the fiber protein [57], and that exposes the adhesin surface at the tip of the LTF. Significantly, the Gp251-Gp254 proteins were all identified as PPAPs, i.e., present in the phage particle, whereas the assembly chaperone, Gp255, was not (Table S3). Therefore, it is very likely that Gp254 is the receptor binding protein of fD1, and that amino acid residues from 520 to 700 are important to the binding specificity.

## 4. Conclusions

Here, we have characterized two phages isolated simultaneously against *Y. pestis* strains KIM D27 and EV76 from an incoming sewage water sample obtained from the City of Turku sewage treatment plant (Turku, Finland). Using the *Y. pestis* strains as enrichment hosts was somewhat risky as the last bubonic or pneumonic plague infections had occurred in Turku almost 300 years earlier, during the outbreak in 1711. However, it has been reported that many *Yersinia* phages can also infect other members of *Enterobacteriaceae*, such as *E. coli*, *Shigella,* and *Salmonella* [9]. For example, the *Yersinia* phage L-413C has been suggested to be an *E. coli* phage. Therefore, although fD1 and fEV-1 were isolated using *Y. pestis*, it was not a likely candidate for a natural host for these phages, since *Y. pestis* has not been encountered in Finland for centuries. On the one hand, alternative hosts could be *Y. pseudotuberculosis* strains or strains of other *Enterobacterales.* Therefore, it was not surprising that we could find several *E. coli* hosts for fD1, especially among laboratory strains. In addition to that, the phage appeared to reproduce much more efficiently in *E. coli* strains than in *Y. pestis* (Figure 3). On the other hand, of the hosts we used for screening, fEV-1 could only infect *Y. pestis* strains. This might be a positive feature, as the phages to be used in phage therapy should only be specific for the target microbes, and not infect the patient’s normal flora.

Lipopolysaccharide (LPS) is an important virulence factor of the pathogenic Gram-negative bacteria including pathogenic members of genus *Yersinia* [58,59,60,61,62,63,64]. It also provides receptor sites for many phages, and changes in LPS structure can affect the sensitivity of a bacterium to a phage. While we were not able to identify the exact nature of the receptors for phages fEV-1 and fD1, the EOP of fEV-1 was significantly lowered when tested against LPS mutants (Table 1 and Figure 2), suggesting that the LPS core heptose and glucose residues are essential constituents of the phage receptor.

On the basis of the morphological characterization, fEV-1 is a dwarf myovirus. Currently, this group of viruses is understudied. Here, we add information on these viruses by determining the host range, latency period, and burst size of fEV-1. The latency period of both fEV-1 and ΦPLPE has been determined to be long. It is possible that dwarf myoviruses, in general, have long latency periods, but current data is very scarce. In addition to characterizing the infection of a novel dwarf myovirus, we provide a comprehensive and quantitative proteomic analysis of such a virus, as such data has been missing to date. Here, we confirm the expression of 33 out of 57 predicted gene products for fEV-1; 20 of which are uncharacterized PPAPs. A more in-depth analysis of these is required to verify their homologues in other dwarf myoviruses, with a potential extension to possibly identifying a protein responsible for causing a long latency. Moreover, two out of five genes lacking homologues in sequence databases are expressed PPAPs. The structure and function of these warrant future studies to verify their role in infection, replication, and virus assembly.

## Figures and Tables

**Table 1:** Bacterial strains used in this study. The phage fEV-1 and fD1 sensitivities of the strains are given as efficiency of plating (EOP) as compared with their original host strains, EV76 and KIM D27, respectively, set to 1. See Section 3.2 for details on lipopolysaccharide (LPS) structures.

Bacterial Strains	Skurnik Lab STORAGE #	EOP of fEV-1	EOP of fD1	Comments	Reference
*Yersinia pestis*					
KIM D27	1418	1	1	Non-pigmented derivative of wild type strain KIM10. Lcr^+^ Pgm^−^ Pst^+^	[20]
KIM D27-ΔwaaQ	5147	10^−4^	1	*ΔwaaQ::nptII* Kan^R^, deep rough LPS missing distal *N*-acetylglucosamine and two distal heptose residues	[21]
KIM D27-ΔwaaE	5149	10^−5^	1	*ΔwaaE::nptII* Kan^R^, deep rough LPS missing two distal heptose residues and the proximal glucose residue	[21]
KIM D27-ΔwaaL	5150	1	1	*ΔwaaL::nptII* Kan^R^, deep rough LPS missing the distal *N*-acetylglucosamine residue	[21]
KIM D27-ΔwabD	5151	1	1	*ΔwabD::nptII* Kan^R^, rough variant LPS missing distal Gal residue	[21]
EV76	1281	1	1	Non-pigmented derivative of wild type strain EV	[22]
*Yersinia pseudotuberculosis*					
Pa 3606	2061	0	0	Serotype O:1b	[23]
204	2069		1	Serotype O:5a	[23]
197	2070	0	0	Serotype O:5b	[23]
151	2073	0	10^−4^	Spontaneous rough derivative of serotype O:4a	[23,24]
YPIII::Δwb	2648	0	0		[21]
PB1::Δwb	2649	0	10^−3^		[21]
*Escherichia coli*					
ME 2881-2	ld 536	0	0	Clinical human isolate	
ME 3128	ld 537	0	0	Clinical human isolate	
ME 2886-2	6588	0	1	Clinical human isolate	
TS 2239-1	6729	0	0	Clinical human isolate	
ME 2861	ld 541	0	0	Clinical human isolate	
ME 2863	ld 542	0	0	Clinical human isolate	
TS 2174	ld 543	0	0	Clinical human isolate	
TS 2757	ld 548	0	0	Clinical human isolate	
ME 2671-1	ld 558	0	0	Clinical human isolate	
ME 2676-1	ld 559	0	0	Clinical human isolate	
ME 2680-1	6589	0	1	Clinical human isolate	
ME 2683-1	6590	0	1	Clinical human isolate	
US 1439	6500	0	0	Clinical human isolate	
KP 1708	6501	0	0	Clinical human isolate	
ME 1658	6503	0	0	Clinical human isolate	
ME 1920	6504	0	0	Clinical human isolate	
US 1769-2	6508	0	0	Clinical human isolate	
1100 (R1 drd-19k-1)	251	0	1	Laboratory strain	[25]
C600 su (lambda cI857)	253	0	1	Laboratory strain	
V517	258	0	1	Clinical isolate	[26]
RY13	449	0	1	Laboratory strain	
LE392 (P1-cml, clr100)	629	0	1	Laboratory strain	
P678-54	630	0	1	Laboratory strain	
LE392	631	0	1	Laboratory strain	
JM103	1247	0	1	Laboratory strain	
PM191	1266	0	1	Laboratory strain	
D21f2	1354	0	0.1	Laboratory strain	
D21	1355	0	1	Laboratory strain	
DH1	1378	0	1	Laboratory strain	
HB101	1389	0	1	Laboratory strain	
C600	1424	0	1	Laboratory strain	
*Klebsiella oxytoca*					
TS 2752	547	0	0	Clinical human isolate	
*Shigella*					
872	707	0	0	Quality control strain	

**Table 2:** Primers used in this work.

Primer	Sequence (5′-3′)	Position in LT992259
fEV1-R	CCTTGCTTGCATTCAGTTCA	1198..1179
fEV1-R2	TGCACCTTCATTTCAAGCAG	580..561
fEV1-R3	GCTGAAGTATCGGCTTCCAG	449..430
fEV1-F	GAAGGAGATAGTGCGCGTTC	37351..37370
fEV1-F2	GTGAAACGCTTGATGCTGAA	38002..38021
fEV1-F3	ACCGCACATTCAACAAAACA	38114..38133
fEV1-F4	TCGCCTTCAGGGTATCAATC	33590..33609
fEV1-R4	TCAAGACCCATTGCACTGAA	34155..34136

## Data Availability

The genome sequences of the *Yersinia* phages fEV-1 and fD1 are available in Genbank under the accession numbers LT992259 and HE956711, respectively. The mass spectrometry data have been deposited to the ProteomeXchange [47] consortium via the MassIVE partner repository (https://massive.ucsd.edu/ (accessed on 14 July 2021)) with the dataset identifiers PXD026811 (fEV-1) and PXD026812 (fD1).

## Supplementary Materials

The following are available online at https://www.mdpi.com/article/10.3390/v13071384/s1, Figure S1: Restriction digestion analysis of the fEV-1 genomic DNA, Figure S2: Determination of the physical termini of the phage fEV-1 genome, Table S1: The predicted sigma70 promoters of phage fEV-1, Table S2: Annotation of the fEV-1 genome and PSI-BLAST results for fEV-1 gene products, Table S3: Annotation of the fD1 genome and PSI-BLAST results for fD1 gene products.
