# Supplementary material for: Bacteriophages fEV-1 and fD1 Infect Yersinia pestis
Source: Viruses. 2021 Jul 16;13(7):1384. doi: 10.3390/v13071384 (PMC8309999; doi:10.3390/v13071384)
Supplement: Supplementary file 1 [file viruses-13-01384-s001.zip › viruses-1290658-supplementary.pdf]

viruses

MDPI

Supplementary Materials

Bacteriophages fEV-1 and fD1 infect *Yersinia pestis*

Mikael Skurnik, Salla Jaakkola, Laura Mattinen, Lotta von Ossowski, Ayesha Nawaz, Maria I. Pajunen and Lotta J. Happonen

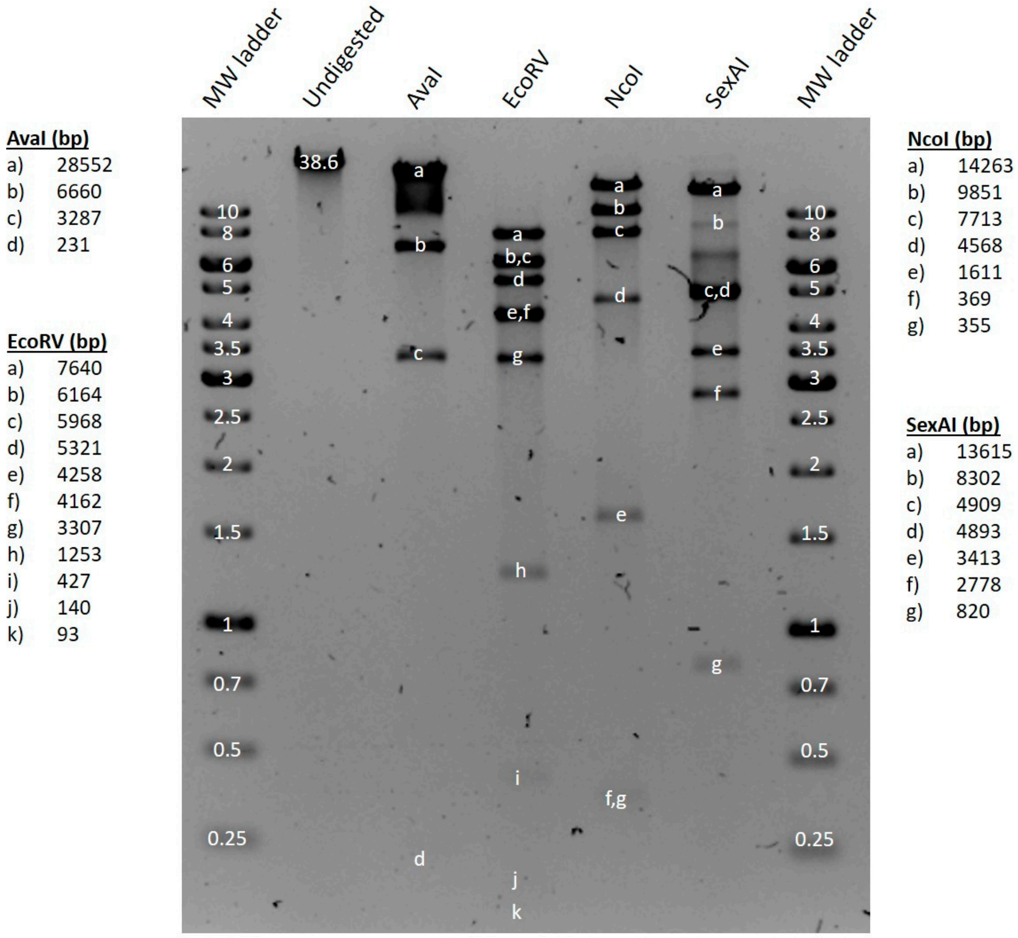

Figure S1. The restriction digestion analysis of the fEV-1 genomic DNA with AvaI, EcoRV, NcoI, and SexAI. The predicted sizes of the restriction fragments based on a circular genome are indicated on both sides of the gel. Notice the presence of partially digested DNA in the AvaI and SexAI lanes.

fEV1 PhageTerm Analysis

UNKNOWN

?

PhageTerm Method

Ends	Left (red)	Right (green)	Permuted	Orientation	Class	Type
Redundant	Random	Random	Yes		-	-

Strand	Location	T	pvalue
+	15599	0.06	7.60e-01
	34864	0.06	3.45e-01
	31509	0.06	3.45e-01
	33641	0.06	1.00e+00
	22582	0.06	1.00e+00
-	34896	0.06	1.00e+00
	19403	0.05	2.60e-01
	23032	0.05	8.66e-01
	33852	0.05	1.00e+00
	25228	0.05	8.66e-01

T (Start. Pos. Cov. / Whole Cov.)

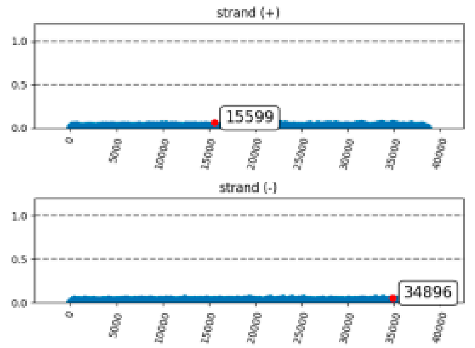

Li's Method

Packaging	Termini	Forward	Reverse	Orientation
OTHER	Absence	No Obvious Termini	No Obvious Termini	NA

Strand	Location	SPC	R
+	21230	15	1.0
	15599	15	-
	4365	14	-
	36877	13	-
	34864	13	-
-	19403	15	1.0
	1559	15	-
	25228	13	-
	23032	13	-
	21676	13	-

SPC

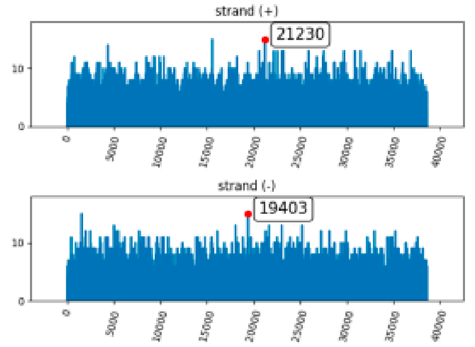

Figure S2. Determination of the physical termini of the phage fEV-1 genome. Failure of the PhageTerm to identify the genome termini.

Table S1. The predicted sigma-70 promoters of phage fEV-1.

Prom	Next gene	Sequence upstream of the start codon of the gene	LDF	-35 box score	-10 box score
P1	g55	TGCGCGTTCTGTATCTGAAACCTGCCCCCCCGCTGAAAAATGCTTGACTATTGATGATTTGAGTACATACAATGCACAAAGGTATCAGGAGGGTTATTTA	3.40	61	47
P2	g33	TCGAACGCGGCGTGCCGCCTGGGGTGTATTACTATCAACTTGAAAACATTGACGGTGTGACTGCAACAGGTGTTCTGTCAGCATACTGGGAGGAACGACC	2.09	60	27
P3	g42	ATCACCACCAATGACCTGGAAGTGAACTTTTTTTAAAGCGGTTGCCTTTTGTGGTACGTTGTGCAACAATACGCATACATTCAACAAAACGGGAACTGAA	3.35	56	36
P4	g47	TAGACATATGAAACCTAATAAATTCCTCAAAGGTCGATCTGGAATATACGGCATTCGCAACTTGGTCAACGATAAAATATACATCGGTAAAACTAAGTGC	2.00	4	71
P5	g52	GCCGTTGATAATCATTGACACAACAAATCCATGCTTGACAGCATACCAGAATTGTTTAACAATGTGCCACGTAGTACAACAAACCAGCGAGGAATAAACC	3.27	66	21
P6	g53	GCACATTGTTAAACAATTCTGGTATGCTGTCAAGCATGGATTTGTTGTGTCAATGATTATCAACGGCTATCATCCGCTATCAACGGCTAAGGAGCGTCCA	3.02	39	67

Table S2. Annotation of the genome of *Yersinia* phage fEV-1.

fEV-1							PSI-BLAST search results [2]				
**Gene**	**Putative Function**	**PPAP [1]**	**Range**	**Strand**	**MW (kDa)**	**Length (aa)**	**Acc. ID**	**Annotation**	**Organism**	**Score**	**E-value**
**g01**	Pentapeptide repeat-containing protein	PPAP	38297:514	+	30.0	279	ECC5451575.1	Pentapeptide repeat-containing protein	*Salmonella enterica*	122	4×10^{-33}
**g02**	Hypothetical protein		504:794	+	10.0	96	-	-	-	-	-
**g03**	Phage terminase, small subunit	PPAP	796:1371	+	21.0	191	WP_206047523.1	Terminase small subunit	*Halomonas venusta*	268	2×10^{-88}
**g04**	Phage terminase, large subunit		1368:2756	+	51.7	462	CAB4157474.1	Putative large terminase	Archaeophage PsiM2	637	0.0
**g05**	Portal protein	PPAP	2753:3922	+	43.8	389	AUR91016.1	Portal protein	Vibrio phage 1.154.O._10N.222.52.B12	382	4×10^{-127}
**g06**	Head morphogenesis protein	PPAP	3915:4715	+	30.2	266	AUR84233.1	Head morphogenesis domain protein	Vibrio phage 1.052.A._10N.286.46.C3	283	2×10^{-92}
**g07**	Hypothetical protein	PPAP	4772:5884	+	40.5	370	YP_007674345.1	Hypothetical protein VPGG_00036	Vibrio phage VBM1	294	1×10^{-92}
**g08**	Hypothetical protein	PPAP	5887:6348	+	15.7	153	AUR84235.1	Hypothetical protein NVP1052A_06	Vibrio phage 1.052.A._10N.286.46.C3	109	3×10^{-27}
**g09**	Major capsid protein	PPAP	6360:7319	+	34.4	319	AUR84236.1	Major capsid protein	Vibrio phage 1.052.A._10N.286.46.C3	349	1×10^{-116}
**g10**	Hypothetical protein	PPAP	7371:7664	+	10.9	97	-	-	-	-	
**g11**	Hypothetical protein	PPAP	7666:8010	+	12.9	114	YP_007674341.1	Hypothetical protein VPGG_00032	Vibrio phage VBM1	92.4	2×10^{-21}
**g12**	Hypothetical protein	PPAP	8060:8533	+	17.2	157	WP_085071357.1	Hypothetical protein	*Pantoea alhagi*	141	3×10^{-40}
**g13**	Hypothetical protein	PPAP	8530:8874	+	12.8	114	MAB53531.1	Hypothetical protein	Marinobacter sp.	115	8×10^{-31}
**g14**	Hypothetical protein	PPAP	8871:9356	+	17.7	161	MBL1319590.1	Hypothetical protein	Methylophaga sp.	140	3×10^{-39}

**g15**	Hypothetical protein	PPAP	9367:10863	+	53.8	498	WP_145558960.1	DUF3383 domain-containing protein	*Yersinia mollaretii*	601	0.0
**g16**	Hypothetical protein	PPAP	10874:11302	+	15.4	142	MBL1319593.1	Hypothetical protein	Methylophaga sp	161	6×10^{-48}
**g17**	Hypothetical protein	PPAP	11313:11729	+	15.9	138	MBA0943622.1	Hypothetical protein	*Escherichia coli*	123	5×10^{-33}
**g18**	Hypothetical protein		11738:11896	+	6.0	52	WP_180272578.1	Uncharacterised protein	*Yersinia mollaretii*	76.3	5×10^{-17}
**g19**	Hypothetical protein	PPAP	11883:13598	+	60.9	571	EEM3686798.1	Hypothetical protein	*Salmonella enterica*	389	1×10^{-124}
**g20**	Hypothetical protein	PPAP	13595:14374	+	27.8	259	WP_085071364.1	Hypothetical protein	*Pantoea alhagi*	222	3×10^{-68}
**g21**	Hypothetical protein	PPAP	14376:14714	+	13.1	112	WP_145558952.1	Hypothetical protein	*Yersinia mollaretii*	124	3×10^{-34}
**g22**	Hypothetical protein	PPAP	14711:15553	+	30.8	280	WP_145527456.1	Hypothetical protein	*Yersinia frederiksenii*	303	6×10^{-99}
**g23**	Hypothetical protein		15546:16178	+	22.5	210	HAT2746464.1	Hypothetical protein	*Citrobacter farmeri*	256	1×10^{-83}
**g24**	Hypothetical protein	PPAP	16192:16551	+	13.8	119	MBE0469603.1	Hypothetical protein	Methyloprofundus sp	115	2×10^{-30}
**g25**	Hypothetical protein	PPAP	16548:17729	+	42.9	393	MAB53542.1	Hypothetical protein	Marinobacter sp	388	1×10^{-129}
**g26**	Hypothetical protein	PPAP	17726:18451	+	27.7	241	HAH8787749.1	DUF2612 domain-containing protein	*Escherichia coli*	273	3×10^{-89}
**g27**	Putative tail fiber protein	PPAP	18444:20246	+	64.8	600	QMP83030.1	Hypothetical protein	Myoviridae sp	263	5×10^{-76}
**g28**	Hypothetical protein		20298:20414	+	4.1	38	-				
**g29**	Reverse transcriptase		20532:21491	+	38.0	319	NCB03306.1	RNA-directed DNA polymerase	Spirochaetia bacterium	300	3×10^{-97}
**g30**	Hypothetical protein		21476:22081	+	22.6	201	MBE0435221.1	Hypothetical protein	Methylomicrobium sp	83.2	2×10^{-16}
**g31**	Phage holin		22078:22356	+	10.2	92	WP_141128063.1	Phage holin, lambda family	*Pseudomonas fluorescens*	71.6	7×10^{-14}
**g32**	Hypothetical protein		22353:22568	+	7.8	71	NGR07855.1	Hypothetical protein	Bacterium SGD-2	109	2×10^{-29}

**g33**	N-acetylmuramoyl-L-alanine amidase, putative	PPAP	22680:23309	+	22.3	209	WP_119020686.1	N-acetylmuramoyl-L-alanine amidase	Halomonas sp. JS92-SW72	245	3×10^{-79}
**g34**	Hypothetical protein		23299:23562	+	9.9	87	WP_092567721.1	Hypothetical protein	*Aidingimonas halophila*	84.7	3×10^{-19}
**g35**	Hypothetical protein		23516:23764	+	8.9	82	WP_153016708.1	Hypothetical protein	*Halomonas axialensis*	97.1	3×10^{-24}
**g36c**	Hypothetical protein		23739:23936	-	7.6	65	WP_150556912.1	Hypothetical protein	*Pandoraea nosoerga*	64.7	6×10^{-12}
**g37c**	Deoxycytidine triphosphate deaminase (EC3.5.4.30)(dUMP-forming)		23933:24469	-	20.2	178	WP_141391763.1	dCTP deaminase	*Cobetia marina*	157	2×10^{-45}
**g38c**	Hypothetical protein		24460:24711	-	9.2	83	NYT81399.1	Hypothetical protein	*Alcaligenaceae bacterium*	50.4	6×10^{-6}
**g39c**	Hypothetical protein		24704:24823	-	4.5	39	ERS04819.1	Hypothetical protein Q673_06280	Marinobacter sp. EN3	42.7	5×10^{-04}
**g40c**	Hypothetical protein		24820:25194	-	14.2	124	-				
**g41c**	Predicted transcriptional regulator	PPAP	25197:26060	-	32.6	287	WP_035234726.1	Chromosome partitioning protein ParB	*Alcanivorax nanhaiticus*	310	4×10^{-102}
**g42c**	Hypothetical protein		26064:26363	-	10.8	99	WP_134021476.1	Hypothetical protein	*Halomonas xianhensis*	121	3×10^{-33}
**g43c**	DNA helicase, phage-associated	PPAP	26428:27912	-	56.6	494	AUR93211.1	Helicase superfamily 1/2 ATP-binding domain protein	Vibrio phage 1.185.O._10N.286.49.C2	845	0.0
**g44c**	Hypothetical protein		27912:28541	-	23.9	209	CAB4141357.1	Hypothetical protein UFOVP414_10	Uncultured *Caudovirales* phage	269	1×10^{-88}
**g45c**	Hypothetical protein		28529:28822	-	10.7	97	AUR93213.1	VRR-NUC domain protein	Vibrio phage 1.185.O._10N.286.49.C2	121	3×10^{-33}
**g46c**	Hypothetical protein	PPAP	28828:29364	-	20.3	178	QGT52244.1	Ribonuclease H-like domain protein	Vibrio phage MZH0603	216	6×10^{-63}
**g47c**	Hypothetical protein		29354:29929	-	22.8	191	NCD06171.1	Hypothetical protein	*Spirochaetia bacterium*	147	2×10^{-40}

**g48c**	DNA polymerase B region	PPAP	30041:31480	-	55.7	479	CAB4146555.1	Hypothetical protein UFOVP503_34	Uncultured *Caudovirales* phage	583	0.0
**g49c**	Hypothetical protein	PPAP	31534:32253	-	25.0	239	AUR82098.1	Protein of unknown function DUF2815	Vibrio phage 1.021.A._10N.222.51.F9	177	2×10^{-32}
**g50c**	Hypothetical protein	PPAP	32278:33582	-	48.2	434	AUR82099.1	Coil containing protein	Vibrio phage 1.021.A._10N.222.51.F9	403	4×10^{-134}
**g51c**	Hypothetical protein	PPAP	33579:34031	-	17.6	150	-				
**g52c**	Hypothetical protein	PPAP	34028:34204	-	6.8	58	XP_010444284.1	Pyruvate kinase, cytosolic isozyme isoform X3	*Camelina sativa*	35.0	2.7
**g53**	Hypothetical protein		34338:34511	+	6.6	57	SDT10036.1	Hypothetical protein SAMN05216271_3587	*Pseudomonas sabulinigri*	68.2	6×10^{-14}
**g54**	Putative primase	PPAP	34501:37023	+	93.9	840	WP_112054867.1	Bifunctional DNA primase/polymerase	*Halomonas taeanensis*	1092	0.0
**g55**	Hypothetical protein		37462:37716	+	9.5	84	MAD98753.1	Hypothetical protein	Flavobacteriaceae bacterium	56.6	2×10^{-08}
**g56**	Hypothetical protein		37713:38087	+	14.3	124	TDX21640.1	Hypothetical protein DFO67_1348	*Halomonas xianhensis*	131	2×10^{-36}
**g57**	Hypothetical protein		38143:38307	+	6.1	54	MBL4781756.1	Hypothetical protein	*Porticoccaceae bacterium*	62.0	1×10^{-10}

[1] PPAP: phage-associated protein identified by LC-MS/MS; [2] PSI-BLAST at https://blast.ncbi.nlm.nih.gov using default setting against the non-redundant protein sequence database release of 2021/05.

Table S3. Annotation of the genome of *Yersinia* phage fD1.

**fD1**								**PSI-BLAST search results [3]**					
**Gene**	**Putative function [1]**	**PPAP [2]**	**Start**	**End**	**Strand**	**MW (kDa)**	**Length (aa)**	**Acc. ID**	**Annotation**	**Organism**	**Score**	**E-value**	**T4 [4]**
**g001**	Protector from prophage-induced early lysis	PPAP	12	2189	-	82.6	725	QHR63679.1	Protector from prophage-induced early lysis	Escherichia phage teqhal	1484	0.0	Yes

**g002**	Hypothetical protein		2200	2403	-	8.1	67	WP_015983553.1	Hypothetical protein	*Escherichia coli*	135	6×10^{-40}	Yes
**g003**	DNA topoisomerase subunit DNA-dependent ATPase	PPAP	2458	4275	-	68.3	605	WP_015983554.1	DNA topoisomerase subunit DNA-dependent ATPase	*Escherichia coli*	1255	0.0	Yes
**g004**	Hypothetical protein		4345	4605	-	9.3	86	QEG05043.1	Hypothetical protein JK23_00004	*Shigella* phage JK23	179	1×10^{-56}	Yes
**g005**	Hypothetical protein		4611	4982	-	14.0	123	QIN97406.1	Hypothetical protein PhiZZ23_005	*Citrobacter* phage PhiZZ23	144	1×10^{-42}	No
**g006**	Hypothetical protein		4985	5161	-	6.8	58	WP_015995878.1	Zinc ribbon domain-containing protein	*Escherichia coli*	123	1×10^{-35}	Yes
**g007**	mRNA metabolism modulator	PPAP	5164	5589	-	16.7	141	QIN97408.1	mRNA metabolism moderator	*Citrobacter* phage PhiZZ23	289	1×10^{-98}	Yes
**g008**	Modifier of suppressor tRNAs	PPAP	5589	5804	-	8.5	71	AUV61145.1	Modifier of suppressor tRNAs	*Escherichia* phage vB_EcoM-fHoEco02	147	2×10^{-44}	No
**g009**	Hypothetical protein	ppap	5818	6087	-	10.2	89	WP_016059243.1	Hypothetical protein	*Escherichia coli*	185	6×10^{-59}	No
**g010**	Modifier of transcription	PPAP	6185	6676	-	18.3	163	YP_009288377.1	Transcriptional regulator	*Shigella* phage SHBML-50-1	329	9×10^{-114}	Yes
**g011**	Hypothetical protein		6750	7250	-	19.0	166	WP_016059245.1	Hypothetical protein	*Escherichia coli*	341	3×10^{-118}	Yes
**g012**	Hypothetical protein		7261	7764	-	19.9	167	WP_016059245.1	Hypothetical protein AS348_gp127	*Escherichia* phage slur14	345	5×10^{-120}	Yes
**g013**	Exonuclease	PPAP	7828	8511	-	26.0	227	YP_009180784.1	3'-5' exoribonuclease	*Escherichia coli*	470	2×10^{-167}	Yes
**g014**	Hypothetical protein	PPAP	8511	8753	-	9.3	80	WP_015969175.1	Hypothetical protein	*Salmonella enterica*	160	1×10^{-49}	Yes
**g015**	Hypothetical protein		8746	8991	-	9.4	81	WP_080181614.1	Exonuclease	*Yersinia* phage PYPS2T	160	2×10^{-49}	Yes
**g016**	Hypothetical protein		8978	9238	-	10.0	86	AYJ74532.1	Hypothetical protein RB51ORF017	Enterobacteria phage RB51	172	3×10^{-54}	No
**g017**	DNA helicase, phage-associated	PPAP	9245	10564	-	50.0	439	YP_002853972.1	AAA family ATPase	Enterobacteriaceae	903	0	Yes

**g018**	Hypothetical protein		10561	10872	-	12.1	103	WP_016059250.1	Hypothetical protein	Enterobacteria phage RB51	210	2 × 10-68	Yes
**g019**	Anti-sigma factor, putative		10874	11620	-	29.1	248	YP_002853974.1	Hypothetical protein	*Shigella flexneri*	496	5 × 10-177	Yes
**g020**	RNA polymerase ADP-ribosylase		11742	12344	-	23.4	200	EFW4204073.1	RNA polymerase ADP-ribosylase	Enterobacteria phage RB27	417	2 × 10-147	Yes
**g021**	NAD-protein ADP-ribosyltransferase		12341	12964	-	24.3	207	YP_009102226.1	NAD-protein ADP-ribosyltransferase	*Serratia* phage PhiZZ30	427	7 × 10-151	Yes
**g022**	Hypothetical protein		13032	13214	-	7.0	60	QIN97692.1	Hypothetical protein T4p022	*Escherichia* virus T4	125	4 × 10-36	Yes
**g023**	Hypothetical protein	PPAP	13223	13693	-	18.4	156	NP_049637.1	Hypothetical protein F412_gp251	*Escherichia* phage wV7	319	6 × 10-110	Yes
**g024**	Hypothetical protein		13686	13850	-	6.1	54	YP_007004765.1	Hypothetical protein ECML134_023	*Escherichia* phage ECML-134	108	1 × 10-29	Yes
**g025**	Transcription modulator		13847	14050	-	8.1	67	YP_009102498.1	Hypothetical protein	*Escherichia coli*	134	2 × 10-39	Yes
**g026**	Transcription modulator	PPAP	14025	14510	-	18.4	161	WP_015969186.1	Transcription modulator under heat shock	Enterobacteria phage Aplg8	324	1 × 10-111	Yes
**g027**	Hypothetical protein		14519	14851	-	12.3	110	YP_010066079.1	Hypothetical protein RB3_028	*Escherichia* phage RB3	224	8 × 10-74	Yes
**g028**	Hypothetical protein	*ppap*	14851	15057	-	8.2	68	YP_009098414.1	Hypothetical protein	*Escherichia* virus RB14	143	3 × 10-43	Yes
**g029**	Capsid protein	PPAP	15154	15399	-	9.2	81	YP_002854363.1	Hypothetical protein	Enterobacteriaceae	169	5 × 10-53	Yes
**g030**	Hypothetical protein		15416	15625	-	7.9	69	WP_074146361.1	Hypothetical protein KMB99_gp030	Enterobacteria phage T6	142	9 × 10-43	No
**g031**	dCTP pyrophosphatase	PPAP	15825	16343	-	20.2	172	YP_010067184.1	dCTP pyrophosphatase	*Escherichia* phage vB_EcoM_112	361	4 × 10-126	Yes
**g032**	Hypothetical protein		16430	16615	+	6.5	61	YP_009030637.1	Hypothetical protein	Enterobacteriaceae	117	4 × 10-33	No
**g033**	DNA primase		16612	17640	-	39.8	342	WP_015995898.1	Hypothetical protein AVU02_gp166	Escherichia phage slur07	704	0.0	Yes
**g034**	Hypothetical protein	*ppap*	17643	17807	-	5.9	54	YP_009197418.1	Hypothetical protein	Bacteria	105	1 × 10-28	Yes

**g035**	Hypothetical protein		17809	18165	-	13.8	118	YP_009102240.1	Hypothetical protein RB27_035	Enterobacteria phage RB27	240	5×10^{-80}	Yes
**g036**	Hypothetical protein		18167	18268	-	3.3	33	EEV9360713.1	Hypothetical protein	*Escherichia coli*	60.8	7×10^{-11}	Yes
**g037**	Hypothetical protein		18361	18717	-	13.6	118	YP_009102240.1	Hypothetical protein RB27_035	Enterobacteria phage RB27	238	6×10^{-79}	Yes
**g038**	Hypothetical protein	ppap	18719	19345	-	24.5	208	EEV9360713.1	Hypothetical protein AS348_gp100	*Escherichia* phage slur14	429	9×10^{-152}	Yes
**g039**	Hypothetical protein	*ppap*	19345	19638	-	11.0	97	YP_009102240.1	Hypothetical protein ACQ54_gp033	*Escherichia* phage HY01	202	2×10^{-65}	Yes
**g040**	Hypothetical protein	ppap	19699	19956	-	10.2	85	YP_009180811.1	Hypothetical protein ECML134_037	*Escherichia* phage ECML-134	174	6×10^{-55}	Yes
**g041**	Discriminator of mRNA degradation		19958	20140	-	6.9	60	YP_009148484.1	Hypothetical protein	*Bacillus cereus*	120	2×10^{-34}	Yes
**g042**	DNA primase/helicase		20199	21626	-	53.6	475	YP_009102512.1	Helicase	*Escherichia* virus T4	987	0.0	Yes
**g043**	Head vertex assembly chaperone		21775	21978	-	6.1	67	WP_015969199.1	Hypothetical protein	*Shigella sonnei*	92	4×10^{-22}	Yes
**g044**	RecA-like recombination protein	PPAP	21971	23152	-	44.1	393	NP_049654.1	RecA-like recombination protein	Enterobacteria phage RB18	810	0.0	Yes
**g045**	Glucosyl transferase	PPAP	23230	24072	-	32.4	280	EFW0223305.1	RB32ORF041c hypothetical protein	*Escherichia* virus RB14	592	0.0	No
**g046**	dCMP hydroxymethylase	PPAP	24069	24809	-	28.6	246	YP_010066924.1	Deoxycytidylate 5-hydroxymethyltransferase	*Escherichia coli*	512	0.0	Yes
**g047**	Hypothetical protein		24817	24966	-	5.5	49	YP_002854377.1	Hypothetical protein RB27_045	Enterobacteria phage RB27	91.3	6×10^{-23}	Yes
**g048**	Immunity to superinfection protein		24963	25214	-	9.1	83	WP_016059266.1	Superinfection immunity protein	Bacteria	158	2×10^{-48}	Yes
**g049**	Hypothetical protein		25222	25602	-	14.2	126	YP_009102250.1	Hypothetical protein	*Escherichia coli*	260	1×10^{-87}	Yes

**g050**	Hypothetical protein		25613	25849	-	8.1	78	WP_015983592.1	Hypothetical protein D862_gp227	*Escherichia* phage vB_EcoM_ACG-C40	154	5×10^{-47}	No
**g051**	DNA polymerase	PPAP	26030	28726	-	103.6	898	WP_016059267.1	DNA polymerase	*Escherichia coli*	1865	0.0	Yes
**g052**	Translational repressor RegA	PPAP	28805	29173	-	14.6	122	YP_006986599.1	Translational repressor RegA	Bacteria	253	7×10^{-85}	Yes
**g053**	Phage DNA polymerase clamp loader		29175	29738	-	21.4	187	WP_015995700.1	DNA polymerase clamp loader subunit A	*Escherichia coli*	383	2×10^{-134}	Yes
**g054**	AAA family ATPase	PPAP	29740	30699	-	35.8	319	WP_015969209.1	AAA family ATPase	Enterobacteriaceae	665	0.0	Yes
**g055**	Sliding clamp	PPAP	30751	31437	-	24.9	228	WP_015995702.1	Sliding clamp DNA polymerase	*Escherichia coli*	464	4×10^{-165}	Yes
**g056**	RNA polymerase binding protein	PPAP	31493	31882	-	14.7	129	WP_015969211.1	RNA polymerase binding protein	*Escherichia* virus T4	270	3×10^{-91}	Yes
**g057**	Hypothetical protein		31892	32080	-	7.5	62	WP_015983598.1	Hypothetical protein	*Escherichia coli*	130	4×10^{-38}	Yes
**g058**	AAA family ATPase	PPAP	32136	33818	-	63.7	560	NP_049667.1	AAA family ATPase	*Escherichia coli*	1145	0.0	Yes
**g059**	Hypothetical protein		33815	34021	-	8.1	68	WP_015969214.1	Hypothetical protein	*Escherichia* virus RB14	139	2×10^{-41}	Yes
**g060**	Hypothetical protein		34002	34265	-	10.2	87	WP_171921543.1	Hypothetical protein	*Escherichia coli*	178	2×10^{-56}	Yes
**g061**	Endonuclease subunit	ppap	34262	35281	-	39.2	339	YP_002854391.1	Recombination endonuclease subunit	*Yersinia* phage PST	706	0.0	Yes
**g062**	Hypothetical protein		35382	35468	+	3.3	28	WP_016059017.1	No significant hits				No
**g063**	Glucosyl transferase	PPAP	35458	36660	-	46.9	400	YP_009153659.1	Alpha-glucosyl-transferase	*Escherichia* virus RB14	836	0.0	Yes
**g064**	Hypothetical protein		36727	36900	-	6.7	57	YP_010071539.1	Hypothetical protein KMC15_gp063	*Escherichia* phage KIT03	118	1×10^{-33}	Yes
**g065**	Hypothetical protein	PPAP	36904	37107	-	7.9	67	YP_002854395.1	Hypothetical protein KMC13_gp173	Escherichia phage vB_EcoM_IME537	141	2×10^{-42}	Yes

**g066**	Hypothetical protein		37076	37393	-	12.4	105	YP_010071539.	DUF2654 domain-containing protein	*Salmonella enterica*	208	1 × 10-67	Yes
**g067**	Hypothetical protein		37395	37613	-	8.5	72	YP_010071101.1	Hypothetical protein	Bacteria	144	1 × 10-43	Yes
**g068**	RNA polymerase sigma factor		37597	38154	-	21.5	185	EBK7480135.1	Sigma factor	*Escherichia* phage vB_EcoM_112	386	1 × 10-135	Yes
**g069**	Hypothetical protein	PPAP	38233	38502	-	10.8	89	WP_015969225.1	Hypothetical protein	Bacteria	185	5 × 10-59	Yes
**g070**	Hypothetical protein		38499	38714	-	8.1	71	YP_009030673.1	Hypothetical protein AS348_gp070	*Escherichia* phage slur14	144	6 × 10-42	Yes
**g071**	Hypothetical protein	PPAP	38717	39043	-	12.8	108	WP_015995916.1	Hypothetical protein FDJ02_gp066	*Shigella* phage Sf2	218	3 × 10-71	Yes
**g072**	Hypothetical protein		39097	39297	-	7.7	66	YP_009180841.1	Hypothetical protein	*Salmonella enterica*	139	2 × 10-41	Yes
**g073**	Hypothetical protein		39298	39429	-	5.1	43	YP_009618880.1	Hypothetical protein KMC13_gp181	*Escherichia* phage vB_EcoM_IME537	91.7	5 × 10-23	Yes
**g074**	Hypothetical protein		39437	39730	-	11.8	97	WP_015983611.1	Hypothetical protein	*Escherichia coli*	193	5 × 10-62	Yes
**g075**	Hypothetical protein		39723	39905	-	6.9	60	YP_010071109.1	Hypothetical protein	Bacteria	122	7 × 10-35	Yes
**g076**	Hypothetical protein		39930	40016	+	3.1	28	WP_015969231.1	Hypothetical protein RB14ORF72	*Escherichia* virus RB14	55.1	4 × 10-09	No
**g077**	NrdH glutaredoxin		40064	40372	-	11.7	102	WP_100771313.1	NrdH glutaredoxin	Bacteria	211	1 × 10-68	Yes
**g078**	Hypothetical protein		40375	40587	-	7.9	70	YP_002854408.1	Hypothetical protein	Bacteria	137	1 × 10-40	Yes
**g079**	Hypothetical protein		40597	40710	-	4.5	37	WP_015969233.1	Putative ribonucleotide reductase of class III activating protein	*Salmonella* phage pSe_SNUABM_01	78.6	8 × 10-18	No
**g080**	Ribonucleotide reductase of class III (anaerobic)		40703	41173	-	18.1	156	WP_015969234.1	Ribonucleotide reductase of class III (anaerobic), activating protein	*Escherichia* phage ECML-134	323	1 × 10-111	Yes
**g081**	Anaerobic NTP reductase, large subunit	PPAP	41170	42987	-	67.4	605	YP_010075342.1	Anaerobic NTP reductase large subunit	*Escherichia* phage wV7	1256	0.0	Yes

**g082**	Recombination endonuclease VII	PPAP	42984	43457	-	18.2	157	YP_009102550.1	Recombination endonuclease VII	Bacteria	322	4×10^{-111}	Yes
**g083**	Pin protease inhibitor		43499	43984	-	18.8	161	YP_007004820.1	Hypothetical protein KMC04_gp083	*Escherichia* phage vB_EcoM_F1	329	9×10^{-114}	Yes
**g084**	Hypothetical protein		43968	44123	-	6.2	51	WP_015969239.1	Conserved protein of unknown function	*Escherichia* virus T4	105	2×10^{-28}	Yes
**g085**	Hypothetical protein	PPAP	44108	44428	-	12.7	106	YP_010068568.1	Hypothetical protein KMC11_gp082	*Escherichia* phage vB_EcoM_G4507	214	5×10^{-70}	Yes
**g086**	Hypothetical protein		44440	44610	-	6.6	56	NP_049695.1	Hypothetical protein KMC34_gp078	*Shigella* phage SH7	114	4×10^{-31}	Yes
**g087**	Hypothetical protein	*ppap*	44613	44828	-	8.1	71	YP_010070460.1	Hypothetical protein KMC34_gp078	*Shigella* phage SH7	146	2×10^{-43}	No
**g088**	NrdC thioredoxin		44825	45088	-	10.0	87	YP_010076700.1	NrdC thioredoxin	Enterobacteriaceae	182	4×10^{-58}	Yes
**g089**	Hypothetical protein	PPAP	45090	45311	-	8.5	73	YP_010076700.1	Hypothetical protein	Enterobacteria phage RB51	150	7×10^{-46}	Yes
**g090**	Hypothetical protein		45335	45724	-	14.9	129	WP_015969245.1	Hypothetical protein	*Escherichia* virus RB14	268	8×10^{-91}	Yes
**g091**	Thioredoxin, phage-associated	PPAP	45721	46650	-	36.0	309	YP_002854044.1	Thioredoxin	Enterobacteria phage GiZh	635	0.0	Yes
**g092**	Thioredoxin, phage-associated	PPAP	46703	47704	-	38.9	333	YP_002854422.1	Thioredoxin	*Escherichia* phage PE37	672	0.0	Yes
**g093**	Thioredoxin, phage-associated	PPAP	47762	48781	-	39.7	339	ANZ51524.1	Thioredoxin	*Citrobacter* phage PhiZZ23	703	0.0	Yes
**g094**	Thioredoxin, phage-associated		48790	49677	-	33.8	295	YP_010073276.1	Hypothetical protein	*Escherichia coli*	596	0.0	Yes
**g095**	Thioredoxin, phage-associated		49685	50092	-	15.5	135	YP_010065734.1	Thioredoxin	Enterobacteria phage Kha5h	274	7×10^{-93}	Yes
**g096**	Hypothetical protein		50148	50675	-	20.8	175	EGI3119413.1	Hypothetical protein	Enterobacteriaceae	352	2×10^{-122}	Yes
**g097**	Hypothetical protein		50736	51038	-	11.9	100	YP_010066760.1	Hypothetical protein KMC34_gp088	*Shigella* phage SH7	209	4×10^{-68}	Yes

**g098**	Hypothetical protein		51140	52108	-	35.8	322	WP_015995939.1	Hypothetical protein	*Salmonella enterica*	636	0.0	Yes
**g099**	Hypothetical protein		52166	52366	-	7.7	66	YP_010076710.1	Hypothetical protein	*Shigella flexneri*	137	8×10^{-41}	No
**g100**	Hypothetical protein	PPAP	52486	53496	-	38.9	336	WP_016059288.1	Hypothetical protein ACQ28_gp094	*Yersinia* phage PST	701	0.0	Yes
**g101**	Hypothetical protein		53496	53957	-	17.9	153	EFW3029406.1	Hypothetical protein	*Shigella sonnei*	308	9×10^{-10}	No
**g102**	Hypothetical protein	PPAP	53960	54481	-	19.2	173	YP_009153697.1	Hypothetical protein	*Salmonella enterica* subsp. enterica	353	8×10^{-123}	No
**g103**	Hypothetical protein		54488	55021	-	20.7	177	EFX0167658.1	Hypothetical protein KMB91_gp052	*Citrobacter* phage vB_CroM_CrRp10	357	3×10^{-124}	Yes
**g104**	Hypothetical protein	*ppap*	55023	55289	-	10.2	88	EDU9380925.1	Hypothetical protein KMC13_gp215	*Escherichia* phage vB_EcoM_IME537	182	7×10^{-58}	No
**g105**	Hypothetical protein		55291	55476	-	6.9	61	YP_010065158.1	Hypothetical protein RB3_102	*Escherichia* phage RB3	123	2×10^{-35}	No
**g106**	Hypothetical protein		55476	55580	-	4.2	34	YP_010071143.1	Hypothetical protein RB3_103	*Escherichia* phage RB3	65.9	3×10^{-13}	Yes
**g107**	Hypothetical protein		55641	55814	-	6.7	57	YP_009098488.1	Hypothetical protein	*Escherichia coli*	118	2×10^{-33}	Yes
**g108**	Hypothetical protein		55804	55998	-	7.6	64	YP_009098489.1	Hypothetical protein	Bacteria	131	1×10^{-38}	Yes
**g109**	Hypothetical protein		56001	56204	-	7.7	67	WP_015995949.1	Hypothetical protein ACQ28_gp103	*Yersinia* phage PST	129	1×10^{-37}	Yes
**g110**	Hypothetical protein		56204	56392	-	7.2	62	WP_015995740.1	Molybdopterin-guanine dinucleotide biosynthesis protein	*Escherichia coli*	127	7×10^{-37}	Yes
**g111**	Hypothetical protein	ppap	56488	56874	-	14.6	128	YP_009153706.1	Hypothetical protein	Bacteria	259	3×10^{-87}	Yes
**g112**	Lysis inhibition regulator, membrane protein		56871	57164	-	11.1	97	YP_002854065.1	rI membrane protein	Enterobacteria phage RB51	203	7×10^{-66}	Yes
**g113**	Hypothetical protein	PPAP	57177	57389	-	8.3	79	WP_074146478.1	Hypothetical protein	Bacteria	141	2×10^{-42}	Yes

**g114**	Thymidine kinase	ppap	57432	58013	-	21.6	193	WP_015969264.1	Thymidine kinase	*Escherichia coli*	402	2×10^{-141}	Yes
**g115**	Hypothetical protein		58023	58178	-	6.1	51	YP_002854065.1	Hypothetical protein	*Escherichia coli*	100	2×10^{-26}	Yes
**g116**	Hypothetical protein		58206	58382	-	6.5	58	WP_015969266.1	Hypothetical protein	*Escherichia coli*	112	4×10^{-31}	No
**g117**	Hypothetical protein	*ppap*	58379	58585	-	7.9	68	WP_015983644.1	Hypothetical protein RB3_114	*Escherichia* phage RB	114	2×10^{-43}	No
**g118**	Hypothetical protein		58582	58794	-	8.5	70	WP_179226787.1	Hypothetical protein	Enterobacteria phage RB51	148	6×10^{-45}	Yes
**g119**	Macro domain-containing protein	*ppap*	58766	59245	-	17.5	159	WP_179226786.1	Hypothetical protein Sf25_gp241	*Shigella* phage Sf25	327	6×10^{-113}	Yes
**g120**	Valyl-tRNA synthetase modifier	PPAP	59242	59583	-	12.9	113	YP_009098500.1	Hypothetical protein	*Escherichia coli*	231	1×10^{-76}	Yes
**g121**	Transglycosylase domain-containing protein		59576	60121	-	20.7	181	YP_002854071.1	Transglycosylase SLT domain-containing protein	Enterobacteriaceae	371	8×10^{-130}	Yes
**g122**	Site-specific RNA endonuclease		60129	60590	-	18.0	153	AUV63521.1	Site-specific RNA endonuclease	Enterobacteriaceae	318	9×10^{-11}	Yes
**g123**	Hypothetical protein	PPAP	60650	60928	-	10.9	92	WP_015995748.1	Hypothetical protein	*Escherichia coli*	187	6×10^{-60}	Yes
**g124**	Hypothetical protein		60928	61194	-	10.1	88	WP_015969273.1	Hypothetical protein	*Escherichia* virus RB14	180	4×10^{-57}	Yes
**g125**	Hypothetical protein		61187	61408	-	8.3	73	WP_016054150.1	Hypothetical protein	*Escherichia coli*	152	1×10^{-46}	Yes
**g126**	Hypothetical protein		61408	61770	-	13.8	120	WP_015995955.1	Autonomous glycyl radical cofactor GrcA	*Bacillus cereus*	246	3×10^{-8}	Yes
**g127**	Hypothetical protein		61778	62107	-	12.8	109	YP_002854457.1	Hypothetical protein F412_gp150	*Escherichia* phage wV7	226	1×10^{-74}	Yes
**g128**	Hypothetical protein		62104	62643	-	20.3	179	WP_015983654.1	Hypothetical protein e112_126	*Escherichia* phage vB_EcoM_11	372	2×10^{-130}	Yes
**g129**	Head protein	PPAP	62785	63258	-	17.8	157	WP_015983655.1	Hypothetical protein	Enterobacteriacea	320	2×10^{-11}	Yes

**g130**	DenV endonuclease V, N-glycosylase UV repair enzyme		63268	63684	-	16.3	138	YP_007004866.	Pyrimidine dimer DNA glycosylase/endonuclease V	*Escherichia coli*	286	2×10^{-97}	Yes
**g131**	T4-like phage baseplate hub and tail lysozyme	PPAP	63744	64238	-	18.1	164	YP_009030731.1	Glycoside hydrolase family protein	*Escherichia coli*	331	2×10^{-114}	Yes
**g132**	NudE nudix hydrolase	PPAP	64276	64716	-	17.0	146	WP_015983658.1	Nudix hydrolase	*Escherichia* phage vB_EcoM_ACG-C40	308	4×10^{-106}	Yes
**g133**	Hypothetical protein		64713	65201	-	18.9	162	WP_171921338.1	Hypothetical protein	*Escherichia coli*	327	5×10^{-113}	Yes
**g134**	Hypothetical protein		65198	65560	-	14.1	120	EFD8442378.1	Hypothetical protein KMC04_gp133	*Escherichia* phage vB_EcoM_F1	241	2×10^{-80}	Yes
**g135**	Hypothetical protein		65542	65934	-	14.6	130	YP_006986679.1	Conserved hypothetical, predicted membrane protein	*Escherichia* virus T4	243	7×10^{-81}	Yes
**g136**	Hypothetical protein	PPAP	65903	66511	-	23.9	202	EFD7933955.1	Hypothetical protein JB75_0139	*Escherichia* phage vB_EcoM_JB75	412	4×10^{-145}	Yes
**g137**	Hypothetical protein	PPAP	66553	67146	-	22.1	197	YP_010068618.1	Hypothetical protein	*Escherichia coli*	384	2×10^{-134}	Yes
**g138**	Hypothetical protein		67190	67366	-	6.5	58	NP_049740.1	Hypothetical protein RB14ORF134	*Escherichia* virus RB14	119	9×10^{-34}	No
**g139**	Hypothetical protein		67435	67698	-	10.2	87	AXC34057.1	Phage protein	*Escherichia* phage ime09	183	6×10^{-58}	Yes
**g140**	Hypothetical protein	PPAP	67942	68415	-	17.3	157	WP_015969290.1	Hypothetical protein e112_137	Escherichia phage vB_EcoM_112	317	5×10^{-109}	No
**g141**	Hypothetical protein	PPAP	69572	70180	-	22.9	202	VUF55460.1	Hypothetical protein	*Escherichia* phage T4_ev151	404	4×10^{-14}	No
**g142**	Hypothetical protein	PPAP	70280	70795	-	20.0	171	YP_002854470.1	Hypothetical protein KMC31_gp267	*Shigella* phage CM8	343	6×10^{-119}	No
**g143**	Hypothetical protein		70798	71088	-	10.8	96	YP_009197316.1	Hypothetical protein AVU02_gp120	*Escherichia* phage slur07	187	7×10^{-60}	Yes
**g144**	Hypothetical protein	PPAP	71091	71471	-	14.3	126	YP_007004513.1	Hypothetical protein	*Shigella flexneri*	261	4×10^{-88}	Yes

**g145**	Hypothetical protein		71473	71658	-	6.6	61	EEX1215785.1	Hypothetical protein	*Escherichia coli*	117	6 × 10-33	Yes
**g146**	Head protein	PPAP	71727	72011	-	10.1	94	YP_009030742.1	Hypothetical protein KMC01_gp262	*Escherichia* phage EC121	188	4 × 10-60	Yes
**g147**	Hypothetical protein	*ppap*	72084	72539	-	17.1	151	WP_015983675.1	Hypothetical protein	Bacteria	312	2 × 10-107	Yes
**g148**	Chaperone for tail fiber formation	PPAP	72539	72781	-	8.7	80	VUF55460.1	Putative chaperone long and short tail fiber assembly protein	*Shigella* phage Shfl2	151	7 × 10-46	Yes
**g149**	Deoxynucleoside monophosphate kinase	PPAP	72781	73506	-	27.3	241	QEG04817.1	Putative deoxynucleotide monophosphate kinase	*Shigella* phage Shfl2	499	1 × 10-17	Yes
**g150**	Tail completion and sheath stabilizer protein	PPAP	73556	74086	-	19.7	176	YP_009197316.1	Tail completion and sheath stabilizer protein	*Escherichia* phage vB_EcoM_ACG-C40	362	2 × 10-126	Yes
**g151**	DNA end protector protein	PPAP	74193	75017	-	31.5	274	EFW3029441.1	DNA end protector protein	Enterobacteria phage RB18	561	0.0	Yes
**g152**	Head completion protein		75017	75469	-	17.6	150	EEX1215785.1	Head completion	Bacteria	309	3 × 10-10	Yes
**g153**	Baseplate wedge protein	PPAP	75517	76107	+	22.7	196	YP_010067823.1	Baseplate wedge protein	*Escherichia coli*	399	4 × 10-140	Yes
**g154**	Baseplate hub subunit and tail lysozyme		76091	76222	+	4.5	43	WP_015983675.1	Phage tail protein	*Shigella flexneri*	77.4	1 × 10-17	Yes
**g155**	Baseplate hub subunit and tail lysozyme	PPAP	76222	77817	+	58.1	531	YP_004415038.1	Baseplate hub subunit and tail lysozyme	Enterobacteria phage RB51	1084	0.0	Yes
**g156**	Hypothetical protein		77810	78346	+	20.0	178	YP_004415039.1	Hypothetical protein ECML134_147	*Escherichia* phage ECML-134	359	4 × 10-125	Yes
**g157**	PAAR domain-containing protein		78347	78640	+	10.2	97	YP_006986698.1	PAAR domain-containing protein	Bacteria	199	3 × 10-64	Yes
**g158**	Baseplate wedge subunit	PPAP	78649	80631	+	74.4	660	YP_010067029.1	Baseplate wedge subunit	Enterobacteria phage GiZh	1346	0.0	Yes

**g159**	Baseplate wedge initiator	PPAP	80628	83726	+	11.9	1032	WP_015983680.1	Baseplate wedge subunit	*Escherichia coli*	2138	0.0	Yes
**g160**	Baseplate wedge subunit	PPAP	83719	84723	+	37.5	334	WP_016039570.1	Baseplate wedge subunit	Enterobacteria phage GiZh	684	0.0	Yes
**g161**	Baseplate wedge tail fiber connector	PPAP	84787	85653	+	31.0	288	EFW4640011.1	Putative baseplate wedge tail fiber connector	*Shigella* phage Shfl2	587	0.0	Yes
**g162**	Baseplate wedge subunit and tail pin	PPAP	85653	87461	+	65.6	602	YP_002854107.1	Baseplate wedge subunit and tail pin	*Citrobacter* phage vB_CroM_CrRp10	1220	0.0	Yes
**g163**	Baseplate wedge subunit and tail pin	PPAP	87461	88120	+	23.7	219	YP_009102622.1	Phage baseplate wedge	*Escherichia* phage ime09	447	2×10^{-158}	Yes
**g164**	Short tail fibers	PPAP	88117	89700	+	55.8	527	WP_015969311.1	Putative short tail fiber protein	*Shigella* phage Shfl2	1079	0.0	Yes
**g165**	Fibritin	PPAP	89697	91160	+	51.8	487	YP_010066350.1	Hypothetical protein SAJKIND_2	*Staphylococcus* phage SAJK-IND	95	0.0	Yes
**g166**	Neck protein	ppap	91193	92122	+	34.7	309	EFR8158913.1	Putative neck protein	*Bacillus cereus*	640	0.0	Yes
**g167**	Neck protein	ppap	92124	92894	+	29.6	256	YP_010066352.1	Neck protein	*Escherichia* phage vB_EcoM_112	523	0.0	Yes
**g168**	Tail sheath stabilizer and completion protein	PPAP		+		29.6	256	YP_004415050.1	Tail sheath stabilizer and completion protein	*Escherichia coli*	529	0.0	Yes
**g169**	Small terminase protein	*ppap*	93763	94257	+	18.4	164	YP_010065217.1	Small terminase protein	Bacteria	338	4×10^{-117}	Yes
**g170**	Large terminase protein		94241	96073	+	69.7	610	YP_007004536.1	Terminase large subunit	*Escherichia* phage ECML-134	1273	0.0	Yes
**g171**	Tail sheath protein	PPAP	96105	98084	+	71.3	659	YP_004415053.1	Putative tail sheath protein	*Escherichia* phage vB_EcoM_G8	1342	0.0	Yes
**g172**	Tail protein	PPAP	98201	98692	+	18.4	163	ATN93928.1	Phage tail protein	Bacteria	336	2×10^{-116}	Yes
**g173**	Portal protein	PPAP	98776	100350	+	61.0	524	WP_016039581.1	Putative portal vertex of the head	*Shigella* phage Shfl2	1095	0.0	Yes
**g174**	Prohead core protein		100350	100589	+	8.9	79	YP_009030770.1	Prohead core protein	Bacteria	143	9×10^{-43}	Yes

**g175**	Prohead core protein	PPAP	100721	101014	+	11.0	97	WP_016054185.1	Prohead core	*Escherichia* virus RB3	192	3 × 10-61	Yes
**g176**	Prohead core scaffolding protein and protease	PPAP	101014	101652	+	23.3	212	WP_015969323.1	Prohead core scaffolding protein and protease	Bacteria	431	1 × 10-152	Yes
**g177**	Prohead core protein	PPAP	101683	102492	+	29.9	269	YP_009102636.1	Prohead core scaffold protein	*Escherichia* phage vB_EcoM_ACG-C40	525	0.0	Yes
**g178**	Major capsid protein	PPAP	102511	104076	+	56.3	521	YP_010069460.1	Major capsid protein	*Shigella* phage SHFML-11	1065	0.0	Yes
**g179**	Capsid vertex protein	PPAP	104160	105443	+	47.0	427	WP_015969329.1	Capsid vertex protein	*Escherichia coli*	872	0.0	Yes
**g180**	RNA ligase	PPAP	105473	106477	-	37.5	334	YP_004415062.1	RNA ligase	*Escherichia* phage slur14	681	0.0	Yes
**g181**	Hypothetical protein		106487	106765	-	11.0	92	WP_016054188.1	Hypothetical protein	Enterobacteriaceae	188	5 × 10-60	Yes
**g182**	Hypothetical protein		106752	106955	-	7.8	67	YP_803112.1	Hypothetical protein e112_188	*Escherichia* phage vB_EcoM_112	134	2 × 10-39	Yes
**g183**	Capsid protein	PPAP	107057	108475	-	50.6	472	WP_015995787.1	PKD domain protein	*Escherichia* phage vB_EcoM_G10400	916	0.0	Yes
**g184**	Inhibitor of prohead protease	PPAP	108485	109165	-	25.5	226	YP_006986725.1	Inhibitor of prohead protease	*Escherichia* phage vB_EcoM_ACG-C40	454	3 × 10-161	Yes
**g185**	ATP-dependent DNA helicase	PPAP	109216	110727	+	57.9	503	YP_009277546.1	Helicase	*Shigella* phage Sf23	1042	0.0	Yes
**g186**	ATP-dependent DNA helicase	PPAP	110753	110983	+	8.8	76	WP_015995790.1	RNA-DNA and DNA-DNA helicase ATPase	*Escherichia* phage vB_EcoM_112	148	7 × 10-45	Yes
**g187**	Hypothetical protein	PPAP	111039	111206	-	6.1	55	YP_009180676.1	DUF2685 domain-containing protein	Bacteria	112	3 × 10-31	Yes
**g188**	Hypothetical protein		111235	111459	-	9.0	74	WP_015995792.1	Hypothetical protein RB3_183	*Escherichia* phage RB3	154	4 × 10-47	Yes
**g189**	Recombination, repair and	PPAP	111459	111872	-	15.8	137	YP_009030785.1	Recombination, repair and ssDNA-binding protein	*Escherichia* phage ime09	276	9 × 10-94	Yes

	ssDNA-binding protein												
**g190**	Baseplate wedge subunit	PPAP	111939	112337	-	15.1	132	QBO63695.1	GPW/gp25 family protein	Bacteria	267	3×10^{-90}	Yes
**g191**	Baseplate hub subunit	*ppap*	112337	112963	-	23.6	208	YP_006986734.1	gp26 family baseplate hub assembly chaperone	*Escherichia coli*	422	4×10^{-149}	Yes
**g192**	Baseplate hub assembly protein	PPAP	113014	113763	+	28.9	249	YP_010076380.1	Hypothetical protein e112_19	*Escherichia* phage vB_EcoM_112	496	7×10^{-177}	Yes
**g193**	Baseplate hub subunit	PPAP	113763	114938	+	44.4	391	YP_009030789.1	Baseplate hub subunit	*Escherichia* virus RB14	81	0.0	Yes
**g194**	Baseplate hub distal subunit	PPAP	114883	115416	+	20.1	177	WP_015969346.1	Base plate distal hub subunit	Bacteria	357	2×10^{-124}	Yes
**g195**	Baseplate hub subunit, tail length determinator	PPAP	115413	115988	+	19.9	191	YP_009098569.1	Hypothetical protein	*Shigella sonnei*	278	5×10^{-90}	Yes
**g196**	Baseplate hub subunit, tail length determinator	PPAP	116075	117184	+	40.7	369	YP_007004562.1	Hypothetical protein	*Shigella flexneri*	759	0.0	Yes
**g197**	Baseplate tail tube cap	PPAP	117193	118287	+	39.6	364	WP_016039599.1	Baseplate subunit	*Escherichia* virus RB14	740	0.0	Yes
**g198**	Baseplate tail tube initiato	PPAP	118287	119252	+	34.9	321	WP_016059329.1	Hypothetical protein ECML134_192	*Escherichia* phage ECML-134	656	0.0	Yes
**g199**	Hypothetical protein	*ppap*	119281	119571	-	10.7	96	YP_009030796.1	Hypothetical protein	*Salmonella enterica*	189	3×10^{-60}	Yes
**g200**	RNA polymerase ADP-ribosylase	PPAP	119632	121689	-	75.6	685	YP_002854524.1	RNA polymerase-ADP-ribosyltransferase	*Citrobacter* phage PhiZZ23	140	0.0	No
**g201**	RNA polymerase-ADP-ribosyltransferase	PPAP	121693	123786	-	77.6	697	WP_015983717.1	NAD-protein ADP-ribosyltransferase	*Escherichia* phage ime09	1422	0.0	No
**g202**	Hypothetical protein	ppap	123839	124027	-	7.1	62	EFX0168239.1	Hypothetical protein	Bacteria	125	2×10^{-36}	Yes

**g203**	DNA ligase	PPAP	124024	125487	-	55.3	487	EFW3029236.1	DNA ligase	*Escherichia* phage slur14	993	0.0	Yes
**g204**	Hypothetical protein		125484	125753	-	10.8	89	YP_002854527.1	DUF3045 domain-containing protein	*Escherichia coli*	188	2×10^{-60}	Yes
**g205**	Hypothetical protein	PPAP	125753	126589	-	32.3	278	YP_009102667.1	Hypothetical protein BI058_gp203	*Shigella* phage SHBML-50-1	578	0.0	Yes
**g206**	Hypothetical protein		126586	127044	-	17.1	152	WP_016054208.1	Hypothetical protein JK38_00196	*Shigella* phage JK38	313	8×10^{-108}	Yes
**g207**	Hypothetical protein		127037	127243	-	8.1	68	YP_010065838.1	Hypothetical protein	*Escherichia coli*	140	6×10^{-42}	Yes
**g208**	Hypothetical protein		127240	127437	-	7.2	65	YP_007004573.1	Hypothetical protein	Enterobacteria phage RB51	130	7×10^{-38}	Yes
**g209**	Hypothetical protein		127437	127724	-	10.8	95	WP_016039609.1	Hypothetical protein RB3_203	*Escherichia* phage RB3	199	1×10^{-64}	Yes
**g210**	Hypothetical protein		127765	128130	-	14.2	121	YP_009180698.1	Hypothetical protein KMC16_gp211	*Escherichia* phage vB_EcoM_Lutter	149	2×10^{-44}	Yes
**g211**	Hypothetical protein		128199	128531	-	12.9	110	WP_015983724.1	Conserved hypothetical protein	*Escherichia* virus T4	225	3×10^{-74}	Yes
**g212**	Hypothetical protein	PPAP	128642	128818	-	6.5	58	YP_009288565.1	Hypothetical protein ACQ28_gp199	*Yersinia* phage PST	112	5×10^{-31}	Yes
**g213**	Lysis inhibition accessory protein	PPAP	129064	129312	-	9.3	82	QEG06046.1	Hypothetical protei	Bacteria	166	8×10^{-52}	Yes
**g214**	Co-chaperone GroES	PPAP	129460	129795	-	12.1	111	WP_015995813.1	co-chaperone GroES family protein	Bacteria	222	5×10^{-73}	Yes
**g215**	Hypothetical protein	ppap	129852	130160	-	11.5	102	YP_002854159.1	DUF2693 domain-containing protein	*Salmonella enterica*	206	6×10^{-67}	Yes
**g216**	Hypothetical protein		130161	130397	-	9.4	78	YP_009098589.	Tail fiber protein	*Shigella* phage CM8	159	4×10^{-49}	Yes
**g217**	Deoxycytidylate deaminase	PPAP	130397	130978	-	21.3	193	YP_010071953.1	Putative deoxycytidylate deaminase	*Shigella* phage Shfl2	400	6×10^{-141}	Yes
**g218**	Hypothetical protein		130975	131313	-	12.8	112	NP_049822.1	Hypothetical protein Shfl2p216	*Shigella* phage Shfl2	229	1×10^{-75}	Yes
**g219**	Hypothetical protein		131310	131546	-	8.8	78	YP_009153807.1	Hypothetical protein	Enterobacteriaceae	156	4×10^{-48}	Yes
**g220**	Hypothetical protein		131540	132067	-	20.2	175	WP_015969374.1	Hypothetical protein KMC13_gp056	*Escherichia* phage vB_EcoM_IME537	349	2×10^{-121}	No

**g221**	Hypothetical protein		132130	132405	-	10.1	91	WP_015969375.1	Conserved hypothetical protein	*Escherichia* virus T4	185	5×10^{-59}	Yes
**g222**	Hypothetical protein		132408	132608	-	7.9	66	WP_015969376.1	Hypothetical protein KNU25_gp032	*Escherichia* phage MLF4	132	6×10^{-39}	Yes
**g223**	Hypothetical protein		132601	132798	-	7.6	65	YP_010076017.1	Hypothetical protein F413_gp048	*Escherichia* phage ime09	136	3×10^{-40}	Yes
**g224**	AAA family ATPase	PPAP	132798	133706	-	34.9	302	YP_004415106.1	Polynucleotide 5'-kinase and 3'-phosphatase	*Escherichia* phage vB_EcoM_ACG-C40	623	0.0	Yes
**g225**	Hypothetical protein		133703	134023	-	12.1	106	YP_004415107.1	Hypothetical protein D862_gp055	*Escherichia* phage vB_EcoM_ACG-C40	218	1×10^{-71}	No
**g226**	Hypothetical protein		134027	134251	-	8.7	74	WP_015983736.1	Hypothetical protein FDJ03_gp07	*Shigella* phage Sf24	155	1×10^{-47}	Yes
**g227**	Hypothetical protein		134248	134547	-	11.6	99	YP_010070984.1	Hypothetical protein	Bacteria	205	1×10^{-66}	Yes
**g228**	Hypothetical protein		134544	134897	-	13.1	117	NP_049831.1	Hypothetical protein	*Salmonella enterica*	229	9×10^{-76}	Yes
**g229**	Inhibitor of host transcription		134888	135391	-	19.0	167	YP_010099699.1	Hypothetical protein pSs1_00233	*Shigella* phage pSs-1	347	8×10^{-121}	Yes
**g230**	RNA ligase A	PPAP	135456	136580	-	43.5	374	YP_007004594.1	Putative RNA ligase A	*Escherichia* phage vB_EcoM-G28	775	0.0	Yes
**g231**	Endonuclease II		136633	137043	-	15.8	136	YP_006986775.1	GIY-YIG nuclease family protein	Bacteria	280	5×10^{-95}	Yes
**g232**	NrdB aerobic NDP reductase, small subunit	PPAP	137071	137541	-	18.1	156	YP_006986776.	Ribonucleoside-diphosphate reductase 1 subunit beta	*Escherichia* phage vB_EcoM_G8	327	5×10^{-113}	Yes
**g233**	Homing endonuclease		137710	138519	-	30.8	269	YP_009619152.1	HNH endonuclease	*Salmonella enterica*	563	0.0	Yes
**g234**	NrdB aerobic NDP reductase, small subunit	PPAP	138663	139340	-	26.2	225	WP_015983742.1	Ribonucleoside-diphosphate reductase	*Escherichia coli*	466	5×10^{-166}	Yes
**g235**	Ribonucleoside-diphosphate reductase subunit alpha		139392	141656	-	86.0	754	WP_016059118.1	Ribonucleoside-diphosphate reductase subunit alpha	*Escherichia coli*	1576	0.0	Yes

**g236**	Hypothetical protein	*ppap*	141647	141934	-	10.9	95	YP_009111041.	Hypothetical protein	Enterobacteria phage RB51	199	1×10^{-64}	Yes
**g237**	Hypothetical protein		141927	142190	-	10.0	87	YP_010069787.1	Hypothetical protein BI058_gp175	*Shigella* phage SHBML-50-1	178	2×10^{-56}	Yes
**g238**	dTMP (thymidylate) synthase	*ppap*	142187	142693	-	19.7	168	WP_015995830.1	Thymidylate synthetase	Enterobacteria phage RB14	356	2×10^{-122}	Yes
**g239**	Thymidylate synthase		142905	143048	-	4.7	47	YP_010069517.1	Putative thymidylate synthase 2	*Escherichia* phage vB_EcoM_G2540-3	85.9	2×10^{-19}	Yes
**g240**	Hypothetical protein		143094	143441	-	13.7	115	WP_080181527.1	Hypothetical protein ECML134_232	*Escherichia* phage ECML-134	239	2×10^{-79}	Yes
**g241**	Dihydrofolate reductase	PPAP	143462	144043	-	21.6	193	HAJ1134801.1	Dihydrofolate reductase	*Escherichia coli*	401	3×10^{-141}	No
**g242**	Hypothetical protein	PPAP	144043	144288	-	9.7	81	WP_015983747.	Hypothetical protein pSs1_00243	*Shigella* phage pSs-1	165	2×10^{-51}	No
**g243**	Hypothetical protein	*ppap*	144299	144541	-	9.3	80	YP_002854184.1	DUF5417 domain-containing protein	*Escherichia coli*	162	2×10^{-50}	Yes
**g244**	Hypothetical protein	PPAP	144597	144983	-	14.1	128	YP_009288593.1	Hypothetical protein e112_254	*Escherichia* phage vB_EcoM_112	253	7×10^{-85}	Yes
**g245**	Hypothetical protein		145030	145260	-	8.9	76	YP_009210152.1	Hypothetical protein AR1_249	*Escherichia* phage AR1	155	1×10^{-47}	Yes
**g246**	Single-stranded DNA binding protein	PPAP	145406	146314	-	33.5	302	QBO65644.1	ssDNA binding DNA repair recombination and pre-synthesis	Enterobacteria phage GiZh	617	0.0	Yes
**g247**	Hypothetical protein		146414	147067	-	26.0	217	YP_009102707.1	Helicase loading protein	*Escherichia coli*	436	3×10^{-154}	Yes
**g248**	RNA polymerase-associated protein		147064	147402	-	12.8	112	WP_171921485.1	Late promoter transcription accessory protein	Bacteria	223	2×10^{-73}	Yes
**g249**	Double-stranded DNA binding protein	PPAP	147380	147649	-	9.5	89	YP_009111051.1	Double-stranded DNA binding protein	Bacteria	162	9×10^{-50}	Yes
**g250**	RnaseH	PPAP	147658	148575	-	35.6	305	WP_015969401.1	Hypothetical protein	*Escherichia coli*	627	0.0	Yes

**g251**	Phage long tail fiber proximal subunit	PPAP	148680	152549	+	140.0	1289	YP_009030849.1	Long tail fiber proximal subunit	*Citrobacter* phage PhiZZ6	2585	0.0	Yes
**g252**	Phage tail connector protein	PPAP	152570	153685	+	40.3	371	YP_009168060.1	Hypothetical protein FDH37_gp26	*Escherichia* phage HP3	749	0.0	Yes
**g253**	Tail fiber protein	PPAP	153749	154414	+	23.6	221	YP_010066187.1	Putative tail fiber protein	*Escherichia* phage vB_EcoM_WFbE185	449	4×10^{-159}	Yes
**g254**	Long tail fiber, distal subunit	PPAP	154423	157539	+	109.8	1038	WP_171921496.1	Tail fibers protein	*Escherichia* phage mobillu	1947	0.0	Yes
**g255**	Phage tail fibers		157570	158121	+	21.8	183	WP_015969407.1	Putative tail fiber assembly protein	*Escherichia* phage vB_EcoM_G2469	370	3×10^{-129}	Yes
**g256**	Holin lysis mediator		158131	158790	+	24.6	219	WP_015969408.1	Holin	*Escherichia* phage p000v	449	3×10^{-159}	Yes
**g257**	Anti-sigma 70 protein		158791	159063	-	10.5	90	WP_015969409.1	Hypothetical protein HX01_0196	*Escherichia* phage HX01	177	4×10^{-56}	Yes
**g258**	Hypothetical protein		159110	159271	-	6.3	53	QIQ68304.1	Hypothetical protein G2469_00259	*Escherichia* phage vB_EcoM_G2469	102	2×10^{-27}	No
**g259**	Hypothetical protein	PPAP	159261	159560	-	11.6	99	YP_009965878.1	Hypothetical protein	*Shigella flexneri*	202	2×10^{-65}	No
**g260**	Hypothetical protein		159635	159859	-	8.7	74	QBQ77759.1	Hypothetical protein PI26_gp157	*Shigella* phage Shf125875	153	7×10^{-47}	Yes
**g261**	Hypothetical protein		159846	160127	-	10.9	93	QHR72833.1	Hypothetical protein JS09_0193	*Escherichia* phage vB_EcoM_JS09	190	5×10^{-61}	Yes
**g262**	Phage anti-restriction nuclease		160127	160588	-	17.9	153	QBO62946.1	Hypothetical protein JK38_00252	*Shigella* phage JK38	321	6×10^{-111}	No
**g263**	Hypothetical protein		160585	160914	-	12.7	109	AYN56434.1	Hypothetical protein	*Escherichia coli*	227	4×10^{-75}	Yes
**g264**	Activator of middle period transcription	PPAP	160925	161560	-	23.6	211	YP_006907273.1	Activator of middle period transcription	*Escherichia* phage wV7	425	5×10^{-150}	Yes
**g265**	Hypothetical protein		161688	161837	-	4.8	49	QBO62949.1	Hypothetical protein Shfl2p264	*Shigella* phage Shfl2	89.4	4×10^{-22}	Yes
**g266**	DNA topoisomerase	PPAP	161834	163162	-	49.3	442	EGF3885029.1	Topoisomerase II medium subunit	*Escherichia* phage wV7	894	0.0	No

**g267**	Acridine resistance protein		163300	163458	-	5.6	52	YP_009100794.1	Hypothetical protein	Bacteria	102	2×10^{-27}	Yes
**g268**	Ndd nucleoid disruption protein	PPAP	163546	163989	-	16.6	147	YP_009037516.1	Nucleoid disruption protein	*Serratia* phage PhiZZ30	308	7×10^{-106}	Yes
**g269**	Hypothetical protein		164050	164265	-	7.8	71	QEG06102.1	Hypothetical protein	*Escherichia* virus RB14	141	4×10^{-42}	Yes
**g270**	Hypothetical protein		164274	164384	-	4.2	36	WP_179226734.1	Putative outer membrane protein	*Yersinia* phage PST	72.4	9×10^{-16}	Yes
**g271**	Hypothetical protein		164381	164578	-	7.5	65	YP_007005000.1	Hypothetical protein	Bacteria	131	2×10^{-38}	Yes
**g272**	Hypothetical protein		164586	164699	-	4.3	37	YP_004415152.1	Hypothetical protein e112_284	*Escherichia* phage vB_EcoM_112	70.5	1×10^{-14}	Yes
**g273**	Hypothetical protein		164765	164863	-	3.7	32	YP_007005002.1	Predicted outer membrane protein	*Escherichia* phage ime09	65.1	6×10^{-13}	Yes
**g274**	Hypothetical protein		164943	165206	-	10.2	87	WP_015996044.1	Hypothetical protein	*Escherichia coli*	181	1×10^{-57}	Yes
**g275**	DenB DNA endonuclease IV	PPAP	165281	165838	-	21.0	185	YP_010075801.1	Hypothetical protein FT_0153	*Escherichia* phage vB_EcoM_FT	387	5×10^{-136}	Yes
**g276**	Hypothetical protein		165902	166096	-	7.4	64	YP_002854600.1	Hypothetical protein F412_gp002	*Escherichia* phage wV7	127	1×10^{-36}	Yes
**g277**	Protector from prophage-induced early lysis	PPAP	166125	167063	-	35.5	312	YP_009153867.1	Putative protector from prophage-induced early lysis	*Escherichia* phage teqhal	646	0.0	Yes

[1] By similarity to *Escherichia* phage T4, when applicable; [2] PPAP: phage-associated protein identified by LC-MS/MS; ppap: phage-associated protein identified by 2 or more peptides, but with an average spectral count below 2; *ppap*: phage-associated protein identified by 1 peptide only, with an average spectral count of at least 3 and observed in 3 out of 6 sample replicates. [3] PSI-BLAST at https://blast.ncbi.nlm.nih.gov using default setting against the non-redundant protein sequence database release of 2021/06; [4] Similar protein expressed by *Escherichia* phage T4.
